# Functions of miR-146a and miR-222 in Tumor-associated Macrophages in Breast Cancer

**DOI:** 10.1038/srep18648

**Published:** 2015-12-22

**Authors:** Yanshuang Li, Lianmei Zhao, Bianhua Shi, Sisi Ma, Zhenbiao Xu, Yehua Ge, Yanxin Liu, Dexian Zheng, Juan Shi

**Affiliations:** 1 National Laboratory of Medical Molecular Biology, Institute of Basic Medical Sciences, Chinese Academy of Medical Sciences & Peking Union Medical College, Beijing 100005, China; 2 Research center, the Fourth Hospital of Hebei Medical University, Shijiazhuang 050011, China

## Abstract

Tumor-associated macrophages (TAMs) play critical roles in promoting tumor progression and invasion. However, the molecular mechanisms underlying TAM regulation remain to be further investigated and may make significant contributions to cancer treatment. Mammalian microRNAs (miRNAs) have recently been identified as important regulators of gene expression that function by repressing specific target genes mainly at the post-transcriptional level. However, systematic studies of the functions and mechanisms of miRNAs in TAMs in tumor tissues are rare. In this study, miR-146a and miR-222 were shown to be significantly decreased in TAMs associated with the up-regulated NF-κB p50 subunit. miR-146a promoted the expression of some M2 macrophage phenotype molecules, and miR-146a antagomir transfected RAW264.7 monocyte-macrophage cells inhibited 4T1 tumor growth *in vivo*. Meanwhile, overexpression of miR-222 inhibited TAM chemotaxis, and miR-222 in TAMs inhibited 4T1 tumor growth by targeting CXCL12 and inhibiting CXCR4. These data revealed that miRNAs influence breast tumor growth by promoting the M2 type polarization or regulating the recruitment of TAMs. These observations suggest that endogenous miRNAs may exert an important role in controlling the polarization and function of TAMs in breast cancer.

The tumor microenvironment comprises a variety of nonmalignant stromal cells that play pivotal roles in tumor progression and metastasis^1^. Stromal cells include fibroblasts, epithelial cells and many types of immune cells. These cells and the molecules they secrete constitute a local environment critical for cancer development. Among them, tumor-associated macrophages (TAMs) are the most notable migratory immune cells. Evidence from clinical and epidemiological studies has shown a strong association between TAMs density and poor prognosis in several types of cancer, including breast cancer^2^,^3^,^4^,^5^.

Tumor macrophages are heterogeneous cells that respond differently to various micro-environmental signals and display distinct functions. Originally, the commonly held view was that TAMs should have an obvious antitumor effect by killing tumor cells directly or by presenting tumor related antigens to induce the immune response to suppress tumor growth. However, emerging studies have described their functions in other contexts. Many studies showed that TAMs can promote tumor progression and invasion^6^. Furthermore, clinical studies indicate that increased numbers of TAMs are associated with poor prognosis in cancer^7^. However, one view holds that the polarization of macrophages is highly related to tumor stage, indicating that switching from the M1 pro-inflammatory phenotype during the early phase to the M2 anti-inflammatory phenotype occurs and promotes tumorigenesis and progression^8^. TAMs in breast cancer are primarily a macrophage subpopulation with the M2 phenotype^9^. They promote breast cancer progression and metastasis by releasing a variety of cytokines, including chemokines, inflammatory factors and growth factors^9^.

MicorRNAs (miRNAs) are a class of small endogenous 19–24 nt long non-coding RNAs. Mature miRNAs bind to the 3′ UTR of target mRNAs to degrade the mRNA or inhibit the post-transcription processing of target mRNA. They can function at different levels to modulate physiological and pathological processes such as cell division, tumorigenesis, metastasis and the inflammatory response^10^. A number of published studies suggested that miRNAs function in the human monocyte/macrophage response to inflammatory stimuli^11^,^12^,^13^,^14^. However, limited data are available on the systematic expression profile and detailed study of miRNAs in TAMs.

In this study, a transplanted breast cancer mouse model was established and TAMs were isolated to conduct a microRNA microarray. Two significantly down-regulated miRNAs, miR-146a and miR-222, were studied to explore their mechanism and function in breast cancer TAMs. The decreased expression of miR-146a and miR-222 was associated with the up-regulated NF-κB p50 subunit rather than with cytokines in tumor cell culture supernatants. We also found that the inhibition of miR-146a inhibited the expression of M2 macrophage phenotype molecules, and miR-146a antagomir-transfected RAW264.7 monocyte-macrophage cells inhibited 4T1 breast cancer growth *in vivo*. Furthermore, miR-222 inhibited the recruitment of macrophages by targeting CXCL12 and inhibiting CXCR4 to suppress 4T1 tumor growth. These observations suggest that endogenous miRNAs may exert important roles in controlling the polarization and function of TAMs in breast cancer.

## Results

### MiRNA expression profile in TAMs during tumor development

**Figure 1:**
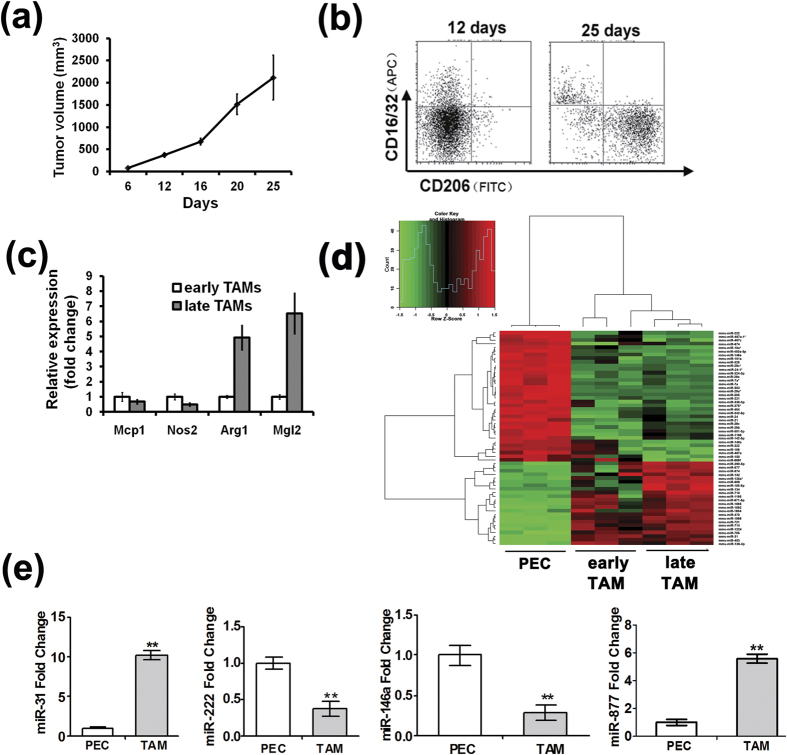
miRNA expression profile in TAMs during tumor development. (**a**) The growth curve of 4T1 tumor in Balb/c mice (n = 5). (**b**) Shift of TAM polarization from the M1 to the M2 subtype during tumor progression were analyzed by FACS (n = 4). (**c**) Total RNA was isolated from the TAMs and expression of Mcp1, NOS2, Arg1 and Mgl2 transcript was determined by qRT-PCR; results expressed as Means ± S.D.; n = 3. (**d**) Heatmap showing expression array data from the miRNA expression screening between PEC and late tumor TAMs (fold changes ≥2 or ≤ 0.5, p ≤ 0.05. Early TAM group refers to TAMs isolated from early 4T1 syngenic tumor (grow to 12 days) tissue. Late TAM group refers to TAMs isolated from advanced 4T1 syngenic tumor (grow to 25 days) tissue. Each sample is biologically duplicated. (**e**) Validation of microarray results by qRT-PCR analysis in PEC and TAM isolated from advanced 4T1 syngenic tumor tissue. U6 snRNA was used as an internal control. Mean ± SD were obtained from three independent experiments. *p < 0.05; **p < 0.01.

Studies have shown that TAMs consist of both M1 and M2 type macrophages. CD16/32 is surface molecules associated with the M1 phenotype, and CD206 is an M2-type molecular surface feature^15^. To study the dynamic microRNA changes in TAMs during tumor development, BALB/c mice were injected subcutaneously with 4T1 cells to establish the 4T1 breast cancer syngenic mouse model (n = 5). As shown in Fig. 1a, the tumor volume in 12 days and 25 days were 372.32 ± 48.85 mm^3^ and 2115.67 ± 505.34 mm^3^, respectively. Cell viability of TAMs isolated was assessed using 7-AAD and as shown in Supplementary Figure 1, cell viability was greater than 85%. TAMs isolated from tumor tissue were analyzed by flow cytometry (FACS) and gated on F4/80^+^CD11b^+^ cells to be further stained with CD16/32 or CD206 antibody to analyze the TAM phenotypes (Supplementary Figure 2). As shown in Fig. 1b, in the TAMs isolated from the mice 12 days after 4T1 cell injection approximately 15.67 ± 3.65% were CD16/32^+^/CD206^−^ M1-type macrophages and 1.23 ± 0.47% were CD16/32^−^/CD206^+^ M2-type macrophages (n = 4). However, in TAMs isolated from the mouse tumors 25 days after 4T1 cell injection, the ratio of the two cell types changed to approximately 1.30 ± 0.92% M1-type cells and 45.70 ± 7.77% M2-type cells (n = 4). Moreover, significantly decreased expression of the M1 markers Mcp1 and Nos2 and significantly increased expression of the M2 markers Arg1 and Mgl2 were also detected in late TAMs (Fig. 1c). These results indicate that a gradual shift in TAMs polarization from the M1 to the M2 subtype occurs during tumor progression, which might be due to the dynamic changes in the tumor microenvironment.

To explore the differences in microRNA expression during TAMs differentiation, TAMs were isolated from the mouse syngenic tumors at different stages. The tumors that formed 12 days after 4T1 cell injection were defined as early tumors, and the tumors that grew for 25 days after 4T1 cell injection were defined as late tumors. MicroRNA expressing profiling of the TAMs was then performed with the Agilent mouse microRNA microarray Rel 12.0. Fold changes greater than 2 or less than 0.5 with a p value smaller than 0.05 were considered differentially expressed. Significantly, there was a pronounced difference in miRNA profiling changes between the control group and the TAM groups. Compared to the peritoneal macrophages (PECs), 23 microRNAs were up-regulated and 36 microRNAs were down-regulated in late tumor TAMs (Fig. 1d, Supplementary Table 1). Similarly, a total of 64 microRNAs were up-regulated and 51 microRNAs were down-regulated in early tumor TAM groups compared with the PECs (Supplementary Table 2). Moreover, 14 microRNAs were down-regulated and 16 microRNAs were up-regulated in late tumor TAMs compared with the early tumor TAMs (Supplementary Figure 3, supplementary Table 3).

In TAM differentiation, the miRNAs which showed similar dynamic expression changes might be related to specific biological functions. Four miRNAs that were differentially expressed between the control group and the advanced tumor TAMs were validated by real-time quantitative RT-PCR (qRT-PCR). The expression of miR-146a and miR-222 significantly decreased and the expression of miR-31 and miR-877 increased in the late 4T1 tumor TAMs compared with PECs (Fig. 1e), consistent with the results of the microarray analysis.

### Down-regulation of miR-146a and miR-222 in TAMs from breast cancer clinical samples

**Figure 2:**
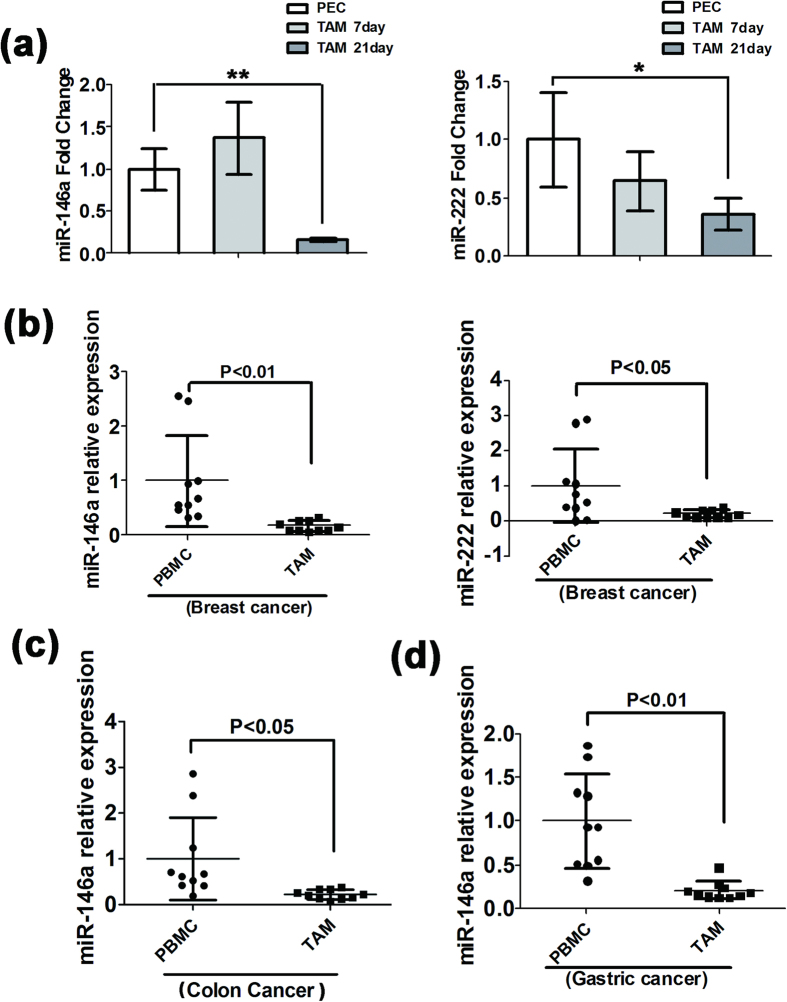
Down-regulation of miR-146a and miR-222 in TAMs from clinical samples. (**a**) The expression levels of miR-146a and miR-222 in TAMs isolated from early 4T1 transplanted tumor tissue (7 days) and late tumor (21 days) compared with PECs via qRT-PCR analysis. U6 snRNA was used as an internal control. Mean ± SD were obtained from three independent experiments. (**b**) Expression levels of miR-146a and miR-222 in TAM isolated from tumor tissue were compared with paired PBMC from breast cancer patients by qRT-PCR assay (n = 10). (**c**) Expression levels of miR-146a in TAM isolated from tumor tissue were compared with paired PBMC from colon cancer patients by qRT-PCR assay (n = 10). (**d**) Expression of miR-146a in TAM isolated from human gastric cancer tissue compared with paired PBMC via qRT-PCR analysis (n = 10). U6 snRNA was used as an internal control. Error bars denote SD; *p < 0.05; **p < 0.01.

We focused on miR-146a and miR-222 because they were the most significant down-regulated miRNAs in late TAMs. To investigate whether there was a dynamic change in miR-146a and miR-222 during TAM developments, TAMs were isolated from 4T1 transplanted tumors grown for 7 days and 21 days. The expression of miR-146a and miR-222 were detected by qRT-PCR. As shown in Fig. 2a, miR-146a expression slightly increased in the early tumor TAMs and significantly decreased in the late tumor TAMs, suggesting that the expression of miR-146a declined with TAM differentiation. The expression of miR-222 slightly decreased in early tumor TAMs and was continuously down-regulated in the late tumor TAMs.

To investigate the expression of miR-146a and miR-222 in TAMs from clinical tumor tissues, miR-146a and miR-222 in paired TAM and peripheral blood monocyte (PBMC) samples isolated from 10 patients with breast cancer were detected by qRT-PCR. TAMs and PBMC isolated were greater than 90% macrophages, assessed by the antibody to human F4/80 (Supplementary Figure 4). As shown in Fig. 2b, miR-146a and miR-222 significantly declined in the patient breast tumor TAMs compared with the paired PBMC, consistent with the result of TAMs in mouse 4T1 transplanted tumor tissue. miR-221, the paralogue of miR-222, showed the same declining trend in mouse 4T1 late tumor TAMs and patient breast tumor TAMs. Meanwhile, miR-31, whose expression increased in mouse 4T1 late tumor TAMs, was also up-regulated in patient breast tumor TAMs compared with paired PBMC (Supplementary Figure 5).

We also tested the expression of miR-146a in the human colon cancer TAMs (Fig. 2c) and gastric cancer clinical samples (Fig. 2d) from ten patients. In agreement with the human breast cancer results, the expression level of miR-146a in TAMs was significantly lower than in the paired PBMC.

### Overexpression of NF-κB p50 in TAMs inhibits miR-146a and miR-222 expression

Studies have shown that miR-146a is up-regulated in LPS-stimulated macrophages and plays a negative regulatory role in the Toll-like receptor-mediated immune response to prevent excessive cell activation^16^,^17^,^18^. miR-222 is elevated during monocytic cell differentiation into macrophages^19^. However, our results showed that both miR-146a and miR-222 decreased in TAMs. Therefore, the reason for the down-regulation of miR-146a and miR-222 in TAMs was investigated. Because there are many cytokines in the tumor microenvironment, including IL-4, IL-6, IL-10, and IL-13, we next asked whether these cytokines down-regulated miR-146a and miR-222 in late tumor TAMs.

**Figure 3:**
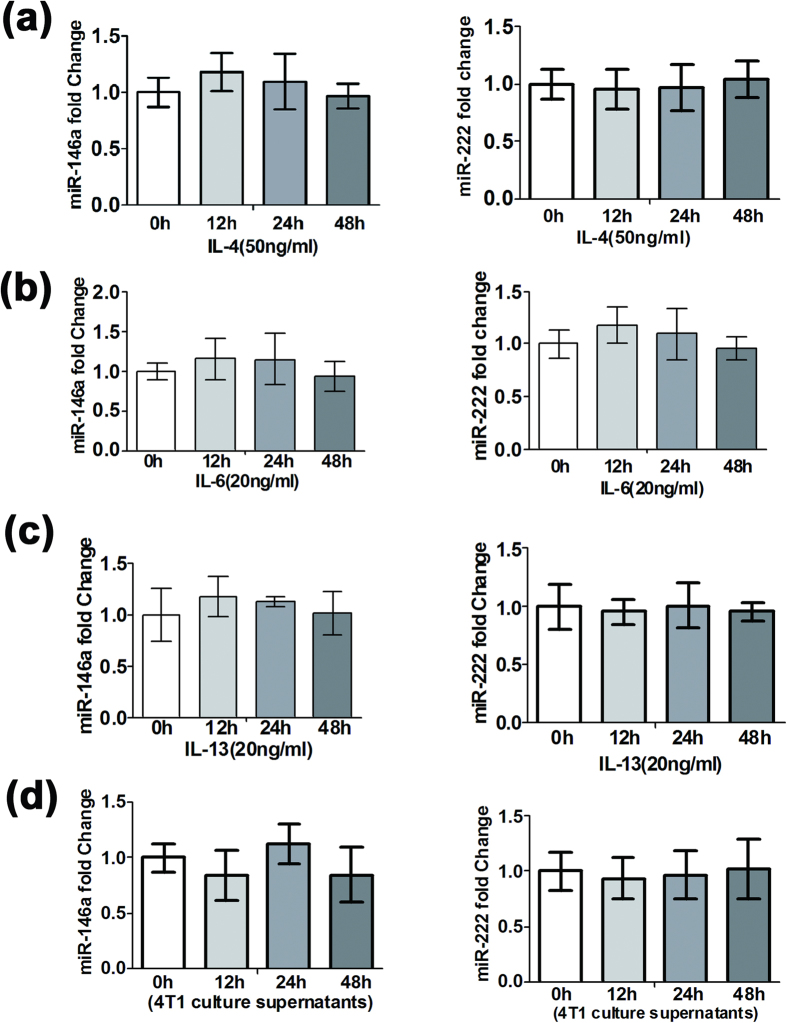
Cytokines and tumor cell culture supernatants had no obvious effect on the expression levels of miR-146a and miR-222. qRT-PCR analysis of the expression levels of miR-146a and miR-222 in PECs under the stimulation of 50 ng/ml IL-4 (**a)**, 20 ng/ml IL-6 (**b**), 20 ng/ml IL-13 (**c**) and 4T1 cell culture supernatant (**d**) for 12 h, 24 h, and 48 h. β-Actin was used as an internal control. Mean ± SD were obtained from three independent experiments.

Mouse PECs were first stimulated with the M2-type macrophage activator IL-4. As shown in Fig. 3a, no obvious changes in miR-146a or miR-222 expression were observed. Then, the mouse PECs were further stimulated with IL-6 or IL-13. The expression of miR-146a and miR-222 detected by qRT-PCR showed that these cytokines did not significantly influence miR-146a and miR-222 expression (Fig. 3b,c). Finally, when the PECs were stimulated with 4T1 cell culture supernatant, miR-146a and miR-222 expression was still not significantly changed (Fig. 3d). Together, these data demonstrated that the main cytokines in the tumor microenvironment and the tumor cell culture supernatant could not change the expression levels of miR-146a and miR-222.

**Figure 4:**
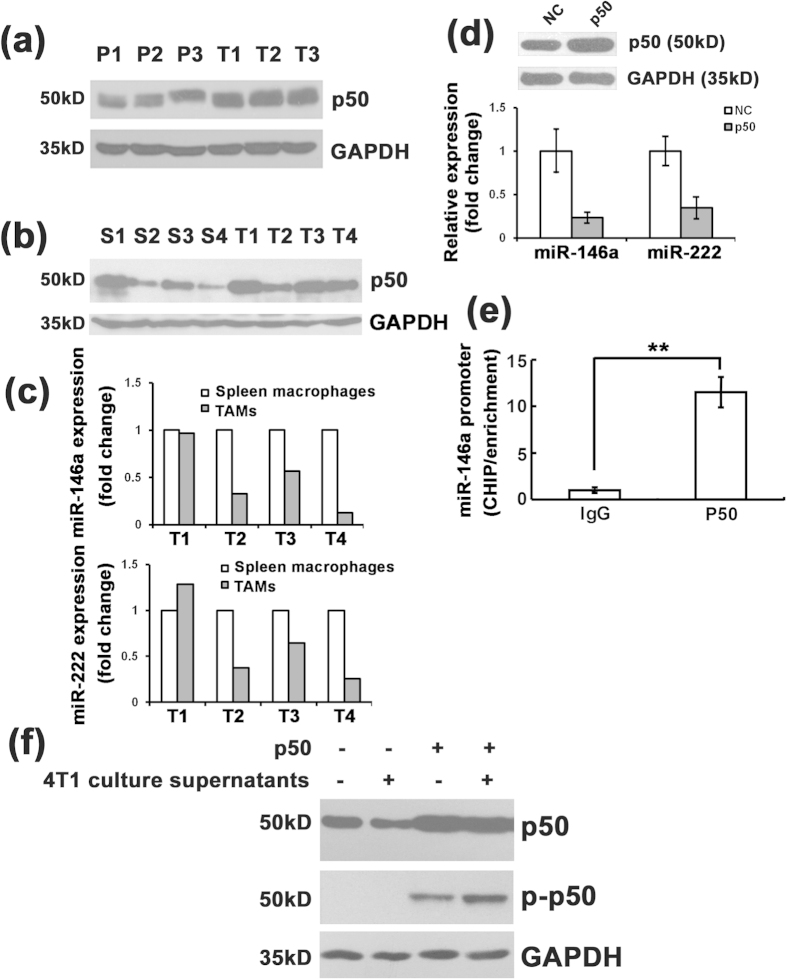
Overexpression of NF-κB p50 inhibited miR-146a and miR-222 expression. (**a**) Western blot analysis of NF-κB p50 expression in TAMs from tumor tissue of breast cancer patients compared with paired PBMCs (P: PBMCs; T: TAMs). (**b**) Western blot analysis of NF-κB p50 expression in TAMs from 4T1 syngenic tumors compared with spleen macrophages (S: spleen macrophages; T: TAMs). (**c**) qRT-PCR analysis of the expression levels of miR-146a and miR-222 in TAMs from 4T1 syngenic tumors compared with spleen macrophages. U6 snRNA was used as an internal control. (**d**) qRT-PCR analysis of miR-146a and miR-222 expression levels in p50 overexpressing RAW264.7 cells. U6 snRNA was used as an internal control. Mean ± SD were obtained from three independent experiments. (**e**) ChIP analysis of the enrichment of the miR-146a promoters bound by p50 or IgG. (**f**) Western blot analysis of NF-κB p50 phosphorylation in RAW264.7 cells with p50 overexpression. Cells were treated with 4T1 conditional medium for 0 or 12 h. **p < 0.01.

The miR-146a promoter region has several NF-κB binding sites, suggesting the NF-κB dependent induction of miR-146a expression^16^. Additionally, two separate distal regions upstream of the miR-222 promoter that are bound by the NF-kB subunit p65 and drive efficient transcription of miR-222 were reported^20^. The NF-κB family consists of five subunits: p65, c-Rel, RelB, p105/p50 and p100/p52. The NF-κB complex is formed by hetero- or homo-dimerization of these different proteins. p65, c-Rel, and RelB are synthesized as mature proteins containing transactivation domains, whereas p105/p50 and p100/p52 are produced as inactive precursor proteins and lack the transactivation domains^21^. Activated NF-κB is generally composed of p65-p50 or c-Rel-p50 heterodimers, whereas homo-dimeric complexes of p50-p50 or p52-p52 are associated with transcriptional repression^22^. Therefore, we speculated that the down-regulation of miR-146a and miR-222 might be mediated by transcriptional repression of NF-κB family members. To this end, a western blot assay was performed to detect the expression of p50 in TAMs in patient breast tumor tissue and paired PBMC. As shown in Fig. 4a, the expression of p50 was significantly higher in breast tumor TAMs compared with PBMC. qRT-PCR analyzing the expression of p50 in TAMs from mouse 4T1 tumors also showed the increased p50 level compared with PECs (Supplementary Figure 6). p50 expression was also elevated in TAMs from 4T1 late tumors compared with macrophages from the spleen of same mouse (Fig. 4b), and its expression was seemed correlated with corresponding miR-146a and miR-222 expression level (Fig. 4c). Next, the p50 overexpression plasmid pwpxl-p50 was constructed and transfected into the murine macrophage cell line RAW264.7. p50 was overexpressed in RAW264.7 cells transfected with pwpxl-p50, and the expression of miR-146a in the p50 overexpressing cells was detected by qRT-PCR analysis. As expected, the miR-146a and miR-222 expression levels were significantly inhibited in p50 overexpressing RAW264.7 cells (Fig. 4d). However, knockdown of p50 with siRNA also decreased the expression of both miR-146a and miR-222 in RAW264.7 cells (Supplementary Figure 7). A ChIP assay was performed to detect whether the p50 subunit could bind to the miR-146a promoter. It indicated that p50 did bind to the miR-146a promoter (Fig. 4e). Because the phosphorylation of p50 is critical for its dimerization and DNA-binding activities^23^, western blot was used to assess the phosphorylation of p50. As shown in Fig. 4f, overexpressed p50 was phosphorylated in p50 overexpressing RAW264.7 cells. Moreover, when cells were treated with 4T1 cell culture supernatant, phosphorylation p50 was increased further.

RelB was a direct target gene of miR-146a as reported^24^, we thus detected the expression of relB in miR-146a overexpression RAW264.7 cells. As shown in Supplementary Figure 8, no obvious change of RelB was observed in miR-146a inhibitor transfected RAW264.7 cells which indicated RelB might not be the target gene in macrophage and there was no feedback loop between RelB and miR-146a.

To analyze whether the p50 repression of the miR-146a and miR-222 expression was specific, we detected the expression of miR-101a and miR-150, which also showed a declining trend in the TAMs. As shown in supplementary Figure 9, no obvious change of miR-101a and miR-150 expression was detected in p50 overexpression RAW264.7 cells.

### Inhibition of miR-146a decreases the expression of M2-type molecules and suppresses tumor growth in mice

**Figure 5:**
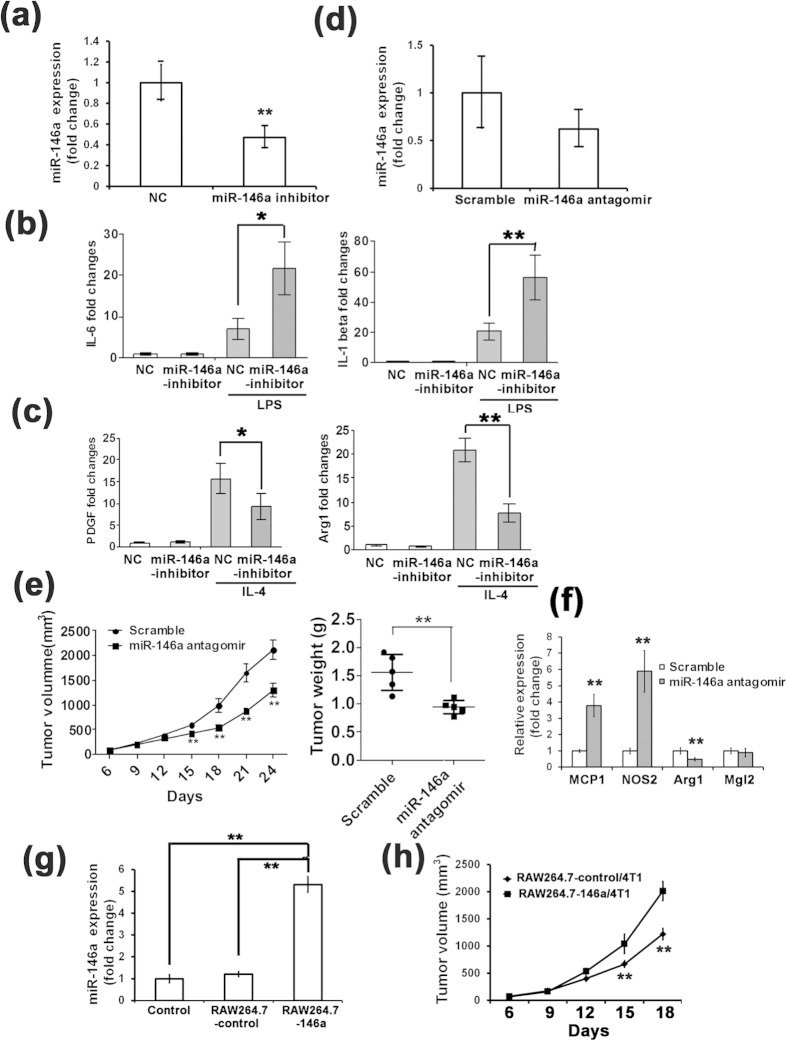
Inhibition of miR-146a decreased some M2 type molecule expression and inhibited tumor growth in mice. (**a**) qRT-PCR analysis of the expression of miR-146a in PECs transfected with the miR-146a inhibitor for 24 h demonstrated the inhibitory efficiency of miR-146a inhibitor in PECs. Mean ± SD were obtained from three independent experiments. (**b**) qRT-PCR analysis of the expression of IL-6 and IL-1β in PECs transfected with the miR-146a inhibitor for 24 h and stimulated by LPS (100 ng/ml) for 12 h compared with the control group transfected with the NC inhibitor. β-Actin was used as an internal control. (**c**) qRT-PCR analysis of the expression of PDGF and Arg1 in PECs transfected with the miR-146a inhibitor for 24 h and stimulated by IL-4 (50 ng/ml) for 12 h compared with the control group transfected with the NC inhibitor. β-Actin was used as an internal control. (**d**) qRT-PCR analysis of the expression of miR-146a in TAMs from 4T1 tumors demonstrated the stable inhibitory efficiency of miR-146a antagomir *in vivo*. Mean ± SD were obtained from the results from 5 mice. (**e**) Down-regulation of miR-146a in macrophages inhibited the growth of 4T1 breast tumor in mice. The syngenic tumor volume and weight in mice subcutaneously injected with RAW264.7 cells transfected with the miR-146a antagomir mixed with 4T1 cells in a ratio of 1:3 were compared with the NC (scramble) group (n = 5). (**f**) Total RNA was isolated from the TAMs and expression of Mcp1, NOS2, Arg1 and Mgl2 transcript was determined by qRT-PCR. n = 4. (**g**) qRT-PCR analysis of miR-146a expression normalized with U6 in RAW264.7-146a cells demonstrated the overexpression of miR-146a in RAW264.7-146a stable cell lines. (**h**) miR-146a overexpression in macrophages promoted the growth of 4T1 breast tumor in mice. The tumor volume in mice subcutaneously injected with RAW264.7-146a cells with 4T1 cells in a ratio of 1:3 were compared with the RAW264.7-control group (n = 5). *p < 0.05; **p < 0.01.

To further investigate the function of miR-146a in TAMs, PECs were transfected with a chemically synthesized miR-146a inhibitor or scramble control (NC) for 24 h and then stimulated with LPS. qRT-PCR was performed to assess the inhibitor efficiency (Fig. 5a). The expression of M1- and M2-type cytokines upon stimulation with LPS or IL-4 was detected by qRT-PCR. Consistent with previous reports, the expression levels of the M1-type molecules IL-6 and IL-1β were increased significantly after transfecting PECs with the miR-146a inhibitor (Fig. 5b). Meanwhile, PECs transfected with the miR-146a inhibitor or NC for 24 h were stimulated with IL-4 to detect the mRNA expression of the M2-type molecules by qRT-PCR. The mRNA levels of PDGF and Arg1 were significantly decreased in the IL-4-stimulated, miR-146a inhibitor transfected PECs (Fig. 5c). However, the expression of the other M2-type molecules Fizz1, Ym1, and CCL17 (Supplementary Figure 10) and the M1-type molecules NOS2, IL-6 and IL-1β did not show obvious change in IL-4-stimulated miR-146a inhibitor transfected PECs (Supplementary Figure 11). These findings suggest that the inhibition of miR-146a promotes the expression of some M1-type molecules and decreases the expression of some M2-type molecules under the indicated stimulation.

To investigate the function of miR-146a in TAMs *in vivo*, RAW264.7 cells were transfected with a miR-146a antagomir or NC, mixed with 4T1 cells in a ratio of 1:3 and inoculated subcutaneously into the back of BALB/c mouse. The antagomir is an artificially synthesized and chemically modified miRNA antagonist. Tumor growth was monitored for 24 days. At the end of the experiment, the animals were euthanized and the tumors were excised and weighed. qRT-PCR study on TAMs isolated from tumors at the end of the experiments demonstrated that the silencing effect of antagomir-146a was long-lasting (Fig. 5d). As shown in Fig. 5e, the tumor growth in terms of both volume and weight was inhibited in miR-146a antagomir transfected macrophages. The increased expression of the M1 markers Mcp1 and Nos2 and significantly decreased expression of the M2 markers Arg1 were observed in TAMs (Fig. 5f), consistent with the *in vitro* results. But another M2 marker, Mgl2, did not changed in TAMs.

RAW264.7 cells were infected with recombinant miR-146a expressing lentivirus, and a stable miR-146a expressing cell line (RAW264.7-146a cells) was established by sorting with a FACS based on the GFP signal from the lentivirus. The miR-146a expression level in the RAW264.7-146a cells was approximately 5-fold higher than in the control cells (Fig. 5g). RAW264.7-146a cells or RAW264.7-control cells were mixed with 4T1 cells in a ratio of 1:3 and injected into the back of BALB/c mice. Tumor growth was monitored by measuring the tumor size for 18 days. As shown in Fig. 5h, RAW264.7-146a cells significantly promoted 4T1 tumor growth. These data demonstrated that miR-146a in TAMs had effect on the growth of 4T1-transplanted tumors in mice.

### miR-222 inhibits macrophage migration *in vitro* and tumor growth *in vivo*

Next, we explored the function and mechanism of miR-222 in TAMs to further clarify its impact on breast cancer progression. PECs were transfected with chemically synthesized miR-222 mimics and inhibitor or NC and then stimulated with LPS to detect the expression level of a panel of cytokines by qRT-PCR (Supplementary Figure 12). Meanwhile, PECs transfected with the miR-222 inhibitor or NC for 24 h were stimulated with IL-4 to detect the mRNA expression of the M2-type molecules IL-10, PDGF, Arg1, Fizz1, Ym1, and CCL17 by qRT-PCR. However, the expression of none of these molecules was varied (Supplementary Figure 13).

**Figure 6:**
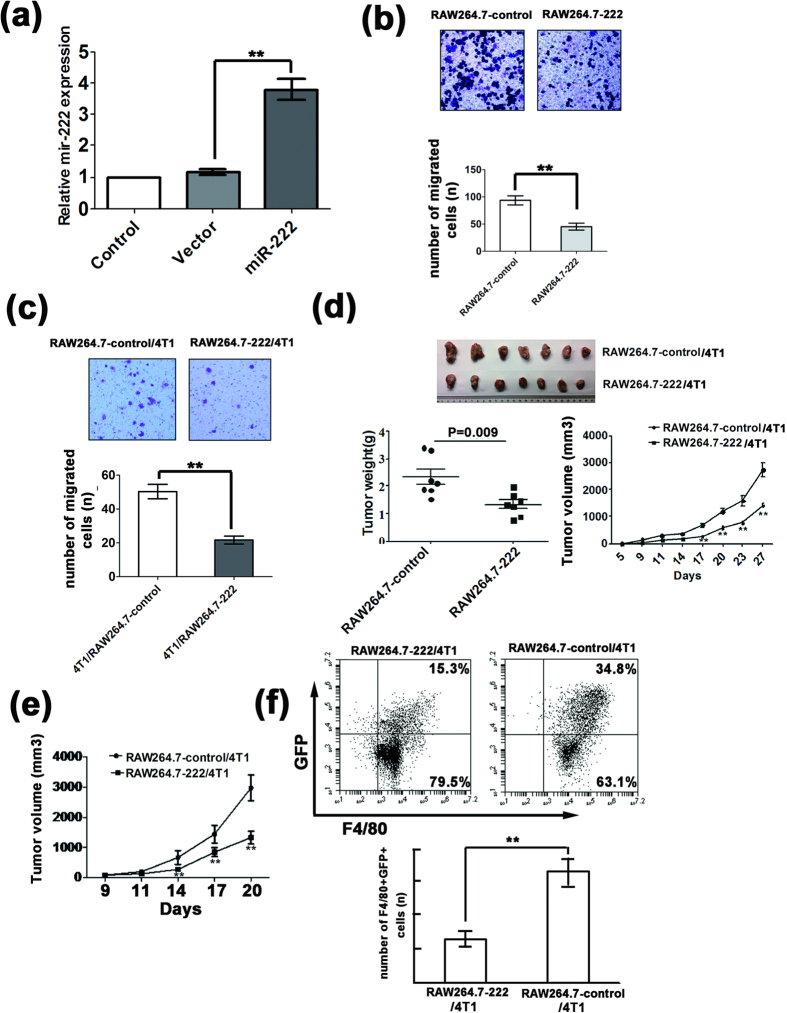
miR-222 inhibited macrophage migration *in vitro* and tumor growth *in vivo*. (**a**) qRT-PCR analysis of miR-222 expression normalized with U6 in RAW264.7-146a cells demonstrated the overexpression of miR-222 in RAW264.7-222 stable cell lines. (**b**) Transwell assay of the migration of RAW264.7-222 cells compared with RAW264.7-control cells. (**c**) Chemotaxis assay of the migration ability of RAW264.7-222 cells towards 4T1 cell culture supernatant compared with RAW264.7-control cells. (**d**) miR-222 overexpression in macrophages inhibited the growth of 4T1 breast tumor in mice. The tumor volume in mice subcutaneously injected with RAW264.7-222 cells with 4T1 cells in a ratio of 1:3 were compared with the RAW264.7-control group (n = 7). RAW264.7-222 cells inhibited 4T1tumor growth, volume and weight compared with vector cells. (**e**) Tail vein injection with RAW264.7-222 cells inhibited the 4T1 subcutaneous tumor growth compared with RAW264.7-control cells (n = 6). 4T1 cells were subcutaneously injected into BALB/c mice. After 3 days, the mice received tail vein injections of RAW264.7-222 cells or RAW264.7-control cells. (**f**) FACS analysis of the F4/80^+^GFP^+^ signal in TAMs isolated from 4T1 tumor tissue from mice received tail vein injections of RAW264.7-222 cells or RAW264.7-control cells. Error bars denote SD; **p < 0.01; n = 4.

Chemoattractant signaling between tumor cells and macrophages is another important function of TAMs^25^. RAW264.7 cells were infected with recombinant miR-222 expressing lentivirus, and a stable miR-222 expressing cell line (RAW264.7-222 cells) was established by sorting with a FACS based on the GFP signal expressed by the lentivirus itself (Supplementary Figure 14). As shown in Fig. 6a, the miR-222 expression level in the RAW264.7-222 cells was approximately 4-fold higher than in the control cells. A transwell assay was performed to determine whether miR-222-overexpressing RAW264.7 cells were involved in the migration of macrophages. RAW264.7-222 cells or empty vector pll3.7-transfected cells (RAW264.7-control cells) were suspended in serum-free RPMI 1640 medium and seeded in the upper chamber. The lower chamber was loaded with RPMI 1640 medium with 20% FBS. As shown in Fig. 6b, the migration ability of RAW264.7-222 cells was obviously decreased compared with the RAW264.7-control cells. RAW264.7-222 cells or RAW264.7-control cells were then seeded in the upper chamber and 4T1 cell culture supernatant was loaded in the lower chamber. The migration ability of RAW264.7-222 cells was obviously weaker in 4T1 culture supernatant compared with the RAW264.7-control cells (Fig. 6c).

To further evaluate the function of miR-222 in TAMs in tumor growth *in vivo*, RAW264.7-222 cells or RAW264.7-control cells were mixed with 4T1 cells in a ratio of 1:3 and injected into the back of BALB/c mice. Tumor growth was monitored by measuring the tumor size for 27 days. As shown in Fig. 6d, miR-222 cells significantly inhibited the tumor growth.

We next asked whether the inhibitory role of miR-222 in TAMs on tumor growth was mediated by affecting macrophage recruitment. To answer this question, 4T1 cells were subcutaneously injected into BALB/c mice. After three days, the mice received tail vein injections of RAW264.7-222 cells or RAW264.7-control cells and tumor volume was measured every three days. As shown in Fig. 6e, tail vein injection with RAW264.7-222 cells significantly inhibited the 4T1 subcutaneous tumor growth. At the end of experiment, we removed and dissociated the tumors and subsequently performed FACS analysis to identify the RAW264.7-222 cells by the GFP signal among F4/80^+^ cells gated on living cells with 7-AAD staining. As shown in Fig. 6f, in the 4T1 tumor tissue from the RAW264.7-222 cell injected mice the F480^+^GFP^+^ signal was significantly weakened compared to the vector cell injected mice, suggesting that miR-222 may inhibit the recruitment of RAW264.7 cells to the tumor.

### CXCL12 is the target of miR-222 in macrophages

**Figure 7:**
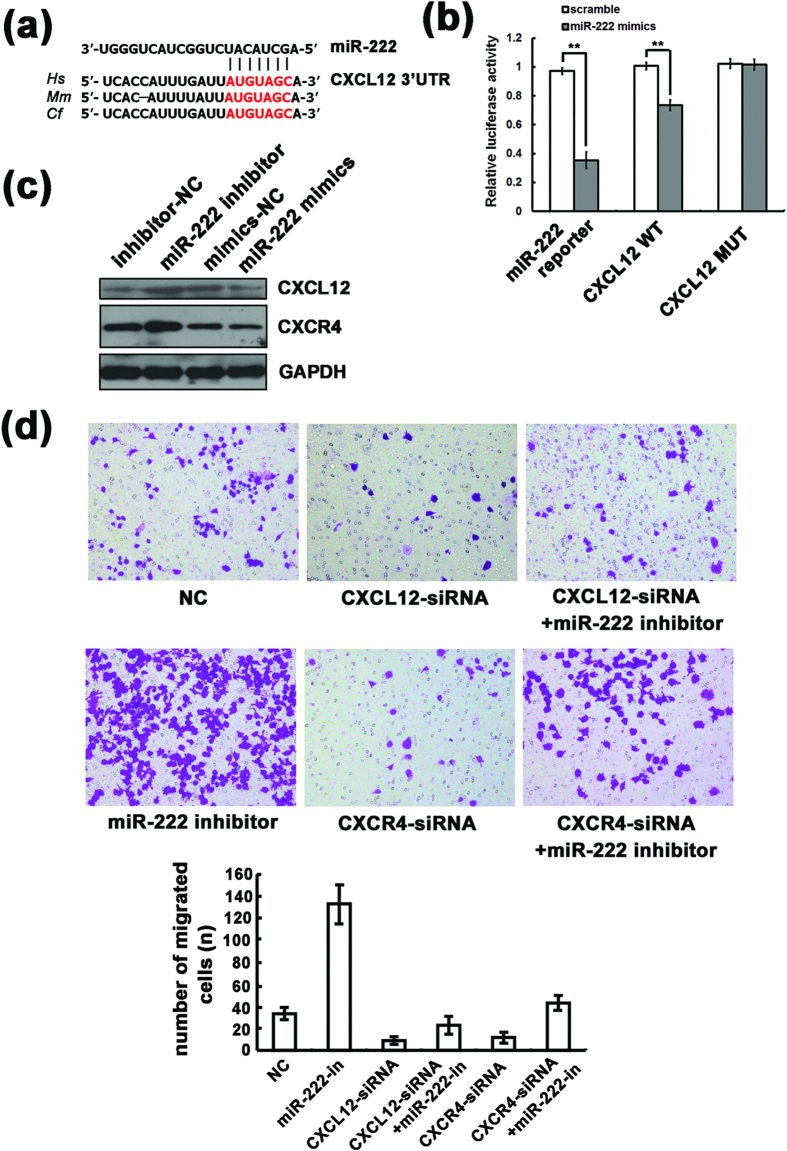
CXCL12 is the target of miR-222 in macrophages. (**a**) Predicted miR-222 binding sites on the CXCL12 3′ UTR. (**b**) Dual-luciferase reporter assay of the relative luciferase activity of the reporter constructs with the positive control (miR-222 reporter), wild type 3′ UTR of human CXCL12 or mutated miR-222 seed binding sites (CXCL12 MUT) co-transfected with scrambled or miR-222 mimics in HEK293 cells. Renilla luciferase activity was normalized to the activity of co-expressed Firefly luciferase. **(c**) Western blot assay of CXCL12 and CXCR4 expression levels in RAW264.7 cells transfected with miR-222 mimics or inhibitor compared with that of cells transfected with NC mimics or NC inhibitor. (**d**) Transwell assay of the migration of RAW264.7 cells transfected with miR-222 inhibitor and siRNAs against CXCL12 or CXCR4. **p < 0.01.

We have shown that the overexpression of miR-222 can inhibit the chemotaxis of macrophages both *in vitro* and *in vivo*. Studies have shown that many chemotactic factors and corresponding receptors contributed to macrophage recruitment to the tumor^26^. The miR-222 targets were analyzed by the commonly used prediction algorithms miRanda and TargetScan. CXCL12 mRNA was a predicted potential target of miR-222 based on the presence of miRNA-binding sites in its 3′ UTR (Fig. 7a). The predicted binding site of miR-222 on the 3′ UTR of CXCL12 was cloned into a luciferase reporter vector. As shown in Fig. 7b, a reporter assay in 293 T cells revealed the miR-222-dependent repression of this 3′ UTR, and mutating the binding site abrogated this reduction in luciferase activity.

RAW264.7 cells were transfected with miR-222 mimics or miR-222 inhibitor, and the cell lysates were subjected to western blot analysis. As shown in Fig. 7c, miR-222 significantly inhibited the protein expression of CXCL12. Conversely, CXCL12 expression increased after endogenous miR-222 was blocked with the miRNA inhibitor. Interestingly, although CXCR4, the receptor of CXCL12, was not a target gene of miR-222 as predicted by bioinformatics analysis, miR-222 showed a similar effect on CXCR4 protein expression. These findings demonstrate that miR-222 targets CXCL12 in macrophages.

To further demonstrate the function of CXCL12 and CXCR4 in miR-222 inhibited macrophage migration, RAW264.7 cells were transfected with siRNAs against CXCL12 or CXCR4. As shown in Supplementary Figure 15 and Fig. 7d, the enhanced migration abilities of the RAW264.7 cells transfected with the miR-222 inhibitor were rescued by knocking down CXCL12 or CXCR4. These data further suggested that the mechanism by which miR-222 exerted its function in macrophage migration might be due to a miR-222/CXCL12 and/or CXCR4 association axis.

## Discussion

To explore the differences in microRNA expression during TAMs differentiation, microRNA expressing profiling of the TAMs was performed with microarray in this study. There was a pronounced difference in miRNA profiling changes between the control group and the early tumor TAM group or late tumor TAMs. We then focused on miR-146a and miR-222 because their greatest down-regulation in late TAMs. Actually, we also compared the miRNA expression between early TAMs and late TAMs (supplementary Figure 2, supplementary Table 3). These miRNAs would be of great interest in order to identify miRNAs involved in macrophage skewing during tumor regression. Further studies in details will be conducted to clarify the function of these miRNAs.

As previously reported, miR-146a was an important miRNA related to the regulation of M1 phenotype macrophages^16^,^17^,^18^. miR-146a is strongly up-regulated upon TLR stimulation and serves as a negative regulator of the MYD88-NF-κB signaling pathway after bacterial infection. miR-146a inhibited the expression of TNF, IL-1β and IL-6 in the THP-1 monocytes tolerant to LPS. Furthermore, artificially overexpressed miR-146a reduced the release of pro-inflammatory cytokines such as CXCL8 and CCL5 in epithelial cells^27^ and IL-6 and CXCL8 in fibroblasts^28^. miR-146a appears to be a negative regulator in the inflammatory response to both bacterial and viral infections. According to our microarray results, miR-146a was the miRNA with the most reduced expression in tumor TAMs. Decreased miR-146a expression was also observed in TAMs from clinical samples of breast cancer, colon cancer and gastric cancer, suggesting that it might be a universal phenomenon in human cancers. We proved that miR-146a inhibition promoted the expression of M1-type molecules in agreement with former reports. We further found that miR-146a inhibition decreased the expression of some but not all M2 type molecules. We attempted to identify the miR-146a target genes in the M2 macrophage signaling pathway but did not get results. Further *in vivo* study conducted in mice suggested that the decreased miR-146a in macrophages inhibited tumor growth, likely consistent with the cytokine variation in macrophages stimulated *in vitro*. There seemed to be somewhat contradiction between the decreased level of miR-146a expression in TAMs and its tumor-promoting function. The miR-146a function in TAMs appeared contradictory to the observation that miR-146a was down-regulated in TAMs. It was reported that miR-146a plays a negative feedback role in Toll-like receptors (TLRs) and the NF-κB signaling pathway^16^,^17^,^18^. Our results suggested that miR-146a might be also a negative regulator in TAM polarization. The physiological role of miR-146a seemed also be to act as a brake in TAMs, similar as the brake in inflammation, thus inhibiting tumor growth.

NF-κB is classically described as a heterodimer composed of the p50 and p65 subunits that drives transcription of a number of inflammatory genes. Saccani *et al.* reported that p50 was up-regulated in TAMs, and NF-κB overexpression accounted for the inability of TAMs to mount an effective M1 antitumor response capable of inhibiting tumor growth^29^. We also found that p50 was up-regulated in TAMs, and overexpression of p50 in macrophages decreased the levels of miR-146a and miR-222. p50 phosphorylation in p50 overexpressing cells further indicated the function of p50 on the decreased miR-146a and miR-222 in TAMs. Moreover, when cells were treated with 4T1 cell culture supernatant, p50 subunit was showed to be more phosphorylated, but no p50 phosphorylation was detected when macrophages treated with 4T1 cell culture supernatant alone, suggesting that cytokine in tumor microenvironment might play an important role together with p50 in miR-146a and miR-222 expression.

However, knockdown of p50 with siRNA decreased the expression of both miR-146a and miR-222 in RAW264.7 cells unexpectedly (supplementary Figure 6). NFκB is generally composed of p65-p50 or c-rel-p50 heterodimers or homodimeric complexes of p50-p50 or p52-p52^21^,^22^. The decreased miR-146a and miR-222 expression in p50 overexpression cells was associated with a predominance of p50-p50 homodimers binding to the putative promoter. But when knockdowned with p50 siRNA, the formation of p65-p50 heterodimer and p50-p50 homodimer might be both inhibited, thus decreasing the miR-146a and miR-222 as result of overall effect.

It was reported that primary blood monocytes secreted the CXCL12 chemokine and expressed the CXCR4 receptor, leading to an autocrine/paracrine loop that shaped monocyte differentiation to a distinct type of macrophage^30^. Schioppa *et al.* found that hypoxia significantly increased the expression of CXCR4 in peripheral blood monocytes, monocyte-derived macrophages, TAMs, endothelial cells, and tumor cells^31^. Analysis of published data suggests that the disruption of the CXCL12-CXCR4 axis could prevent the infiltration of tumors by angiogenic TAM populations. Our results showed that miR-222 was reduced in TAMs, and CXCL12 was identified as a target gene of miR-222, which was not previously reported. Furthermore, miR-222 also inhibited the expression of the receptor of CXCL12, CXCR4. miR-222 inhibited macrophage migration *in vitro* and *in vivo*. Therefore, our results indicated that decreased miR-222 might promote the recruitment of macrophages to the tumor via the CXCL-12/CXCR4 axis.

In this study, we demonstrated two miRNAs, miR-146a and miR-222 were both significantly decreased in TAMs. However, they played contrary role in tumor progression. There is a vast body of literature in cancer research and many signaling pathways have been studied. In some cases, pathways with apparently opposite are involved in the same situation. For example, activation of the ERK subfamily, MAPKs associated with survival, also can be responsible for cell death^32^. Therefore, the involvement of multiple, sometimes conflicting pathways suggests that the cell or organism must be able to integrate diverse and disparate signals into an overall decision.

Taken together, the detailed results in this study indicate new mechanisms by which miRNAs influence breast tumor growth by affecting macrophage polarization or regulating the recruitment of TAMs. These observations suggest that endogenous miRNAs may exert an important role in controlling the polarization and function of TAMs in breast cancer. Therefore, it is possible that miR-146a and miR-222 could become the potentially useful therapeutic target for the treatment of breast cancer.

## Methods

### Cells culture and transfection

The 4T1, RAW264.7 and HEK293 cell lines were obtained from American Type Culture Collection and cultured in RPMI1640 supplemented with 10% fetal bovine serum (FBS) in 5% CO_2_ at 37 °C with saturated humidity. The miR-222 mimics, miR-146a and miR-222 inhibitor were obtained from GenePharma (Shanghai, China). Negative control mimics or inhibitor (Shanghai GenePharma Co., Shanghai, China) were transfected to serve as matched controls. The cells were transfected with mimics, inhibitor or miRNA control at a final concentration of 10 nM using Lipofectamine® LTX with Plus^TM^ Reagent (Life Technologies, MD, USA). According to the manufacturer’s instructionCells were transiently transfected with plasmid or siRNA oligonucleotide against p50, CXCL12 or CXCR4 (Supplementary Table S4, Genepharma, Shanghai, China) using RNAiMax and Lipofectamine® LTX with Plus^TM^ Reagent (Life Technologies, MD, USA).

### Patients and clinical samples

Fresh breast tumor tissue samples and paired peripheral blood were collected from breast cancer 2 and 3 stage patients with tumor resection, fresh gastric and colon tumor tissues and paired peripheral blood were collected from the stage of three and four patients with tumor resection. All human tissue samples were obtained from the Fourth Hospital of Hebei Medical University and were classified according to the standard histological grades of tumors published by the WTO in 2000. Informed consent was obtained from all patients before surgery, and approval was obtained from the ethics committee of the Fourth Hospital of Hebei Medical University (Shijiazhuang, Hebei, China).

### Animal experiments approval

All studies using live mice were performed in strict accordance with the national guidelines and regulations on animal welfare ethics of Institutional Animal Care and Use Committee (IACUC) and were approved by the Beijing Association on Laboratory Animal Care.

### miRNA microarray assay

The tumors formed 12 days after 4T1 cell injection were defined as early tumor, and the tumors grew for 25 days after 4T1 cell injection defined as late tumor. Three group samples of isolated TAMs from early tumors or later tumors and PEC (n = 3 PEC, n = 3 early TAMs, n = 3 late TAMs) were submitted to Shanghai Biotechnology Corporation (Shanghai, China). Total RNA was extracted from each cell sample using TRIzol (Invitrogen, CA, USA) and the PureLink™ miRNA Isolation Kit (Invitrogen, CA, USA) according to the manufacturer’s instructions. RNAs were to be labeled with CyTM5/CyTM3 Mono NHS Ester (Amersham, NJ, USA) using the mirVana miRNA labeling kit (Ambion, TX, USA) and hybridized on the mouse miRNA microarray (v.12.0; Agilent Technologies, CA, USA). Scanning was performed with the axon Scanner Axon GenePix 4000B microarray scanner (Axon Instruments, CA, USA). GenePix Pro V6.0 software (Axon, CA, USA) was used to read the raw intensity of the image. Background subtraction and normalization were performed. The p-value was calculated using the paired t-test. The threshold set for up- and down-regulated genes was a fold change ≥2.0 or ≤0.5 and a p-value ≤ 0.05. Hierarchical clustering was performed to show the distinguishable miRNAs expression pattern among samples using Cluster Treeview software from Stanford University. The profiling data are deposited in NCBI Gene Expression Omnibus and are accessible through GEO series accession number GSE67408.

### Isolation of peritoneal macrophages

Seven weeks old female BABL/c mice were intraperitoneally injected with 2 ml 3% fluid Thioglycollate medium (TG). After three days executed mice and cut the abdominal skin (retain intact peritoneum), was fill the abdominal cavity with PBS containing sodium citrate and pat belly to sucked out the macrophages with a syringe, then centrifuged at 1000 rpm for 10 min. Secondly, the cells were re-suspended with RPMI1640 supplemented with 10% fetal bovine serum, penicillin (100 U/ml), streptomycin (100 μg/ml) and L- glutamine (2 mM) and plated in six-well plates with 1 × 10^6^ cells/ml and cultured for 1.5 h in incubator (5% CO_2_, 37 °C). Finally, medium was discarded and the cells were washed with PBS to remove non-adherent cells. The adherent cells were collected as PEC.

### Animal experiments

For TAMs isolation, six weeks old female Balb/C mice were injected subcutaneously in the right side with 0.1 ml 4T1 cell suspension (concentration of 5 × 10^5^ cells/ml). The tumors were measured with vernier caliper every 3–4 days that the longest diameter as “a” and shortest diameter as “b”, tumor volume was calculated according to the following formula: tumor volume (V) = 1/2 × a × b^2^. When the tumors grow to indicated days, animals were euthanized and tumors were removed.

The effect of miRNAs on growth of 4T1 cells was also examined in this subcutaneous model. Six weeks old female Balb/C mice were injected subcutaneously in the right side with 0.1 ml 4T1 cell suspension (concentration of 5 × 10^5^ cells/ml) or RAW264.7 (transfected with 200 nM of miR-146a antagomir or stable overexpression RAW264.7 cells) cells mixed with 4T1 cells (concentration of 5 × 10^5^ cells/ml) in a ratio of 1:3. At the experimental end point, animals were euthanized and tumors were removed and weighed. For the tail vein injection experiments, six weeks old female Balb/C mice were injected subcutaneously in the right side with 0.1 ml 4T1 cell suspension (concentration of 5 × 10^5^ cells/ml). After three days, the mice were received tail vein injections of 0.1 ml RAW264.7-222 or RAW264.7-control suspension cells (concentration of 1.5 × 10^5^ cells/ml), and tumors’ volume were measured every 3–4 days.

### Isolation of TAMs

After obtaining the fresh tumor tissue, immediately removed the connective tissue, rinsed with RPMI1640 medium, then put into dish sterilized. Cut the tumor into small pieces for 1–2 mm^3^ with ophthalmic scissors, placed in 5 ml digestive RPMI1640 (containing 2% FBS, 0.05% collagenase IV, 0.005% DNase I, had to be 0.22 μm filtered) and transferred to 15 ml centrifuge tube and mixed, 37 °C, 150 rpm acted for 1 to 2 hours. Filtered cell suspension after digestion with 100 mesh screen, good removal of undigested tumor tissue, a repeated filtration, then 50 g, 1 min centrifugal to remove residual tissue. The supernatant was centrifuged at 400 g for 10 min and the precipitate was re-suspended in 2 ml RPMI1640 supplemented with 1% calf serum. Added the cell suspension to 6 cm dishes, which previously added about 4 ml RPMI1640 supplemented with 1% calf serum, and cultured for 1 hour (37 °C, 5% CO_2_). After taking out the cell culture dishes, removed the cell suspension and washed with PBS twice, the adherent cell were TAMs. The cell viability was analyzed by FACS with 7-AAD staining and as shown in Supplementary Figure 1, about 85% isolated TAMs are living1.

### Isolation of human Peripheral blood mononuclear cells

PBMC from patients were isolated by density-gradient centrifugation using Ficoll-Hypaque (Pharmacia, NJ, USA). Briefly, peripheral 20 ml, diluted with three volumes of anticoagulant mixing. Then diluted whole blood was carefully added to the upper layer of separation, 4000 rpm centrifugal 30 min, suck out the buffy coat cells, after washing twice with PBS, 4000 rpm centrifugal 10 min, the supernatant was discarded, resuspended with RPMI1640 (with 10%FBS), plated on a culture dish cultured for 1.5 hour (37 °C, 5% CO_2_). After taking out the cell culture dishes, removed the cell suspension and washed with PBS twice, the adherent cell were PBMC.

### Flow cytometry assay

Flow cytometry was used to analyze the surface markers of macrophages to identify the phenotype of macrophages. Single-cell suspensions were washed in PBS with 2% FCS and adjusted the concentration to 1~5 × 10^6^ cells/ml. For purity analysis of macrophage, cells were incubated with PE-conjugated antibody against mouse F4/80 (eBioscience, CA, USA). For M1 and M2 surface marker analysis, cells were incubated with PE-conjugated antibody against mouse F4/80, PE-Cy5-conjugated antibodies against mouse CD11b (eBioscience, CA, USA), APC-conjugated antibodies against mouse CD16/32 (eBioscience, CA, USA) and FITC-conjugated antibodies against mouse CD206 (Biolegend, CA, USA). All antibodies were used at 5 μg/ml. The cells were incubated with the antibodies for 30 min at 4 °C and washed with PBS. The samples were fixed with 1% paraformaldehyde/PBS and analyzed by using BD Accuri C6 flow cytometer (BD Biosciences, CA, USA). F4/80^+^CD11b^+^ cells were gated and further analyzed for the expression of CD16/32 and CD206. Results were analyzed using FlowJo software (Tree star Inc, Ashland, OR).

### RNA extraction and real-time polymerase chain reaction (qRT-PCR)

For microarray and qRT-PCR analysis, total RNA was extracted from cells using Trizol (Invitrogen, CA, USA) reagent. cDNA was synthesized by M-MLV reverse transcriptase (Invitrogen, CA, USA) from total RNA. Oligo (dT) 18 were used as the RT primers for reverse transcription of mRNAs. For miRNA detection, the reverse transcription of miRNAs was with special primers (supplementary Table 5). qRT-PCR was carried out in BIORAD IQ5 real-time PCR System (Biorad, CA, USA) using SYBR Premix Ex Taq kit (Takara, Dalian, China) according to manufacturer’s instruction. The data were presented as fold change. The comparative Ct method was used to quantify target genes relative to endogenous control. For each individual analysis, control group was designated as the calibrator and given a relative value of 1.0. All quantities were expressed as n-fold relative to the calibrator. The primers used for PCR are listed in supplementary Table 6.

### Western Blot assay

For western blot assay, cells were harvested after transfected for 48 hours and lysed with lysis buffer (1% NP-40, 13.7 μg/ml pepstatin A) and mini protease inhibitor at the ratio of 1:7 for 40 min. Then centrifuged at 12 000 rpm for 20 minutes, the concentrations of supernatant proteins were analyzed using the PierceBCA protein assay kit (Thermo scientific, IL, USA). 40 μg of total proteins were conducted electrophoresis in 12% SDS-PAGE gel, transferred to polyvinylidene difluoride (PVDF) membranes (Millipore) and incubated with 5% TBST skimmed milk powder. The PVDF membranes were incubated with antibodies against phosphorylated p50 (Abcam, MA, USA) and total p50 (Santa Cruz, CA, USA), CXCL12, CXCR4 or GAPDH (Abcam, MA, USA) for 16 h in 4 °C and washed with TBST 10 min for three times. After that we incubated the PVDF membranes with second antibody for 2 hours in room temperature and washed three times for 10 min each. Finally, immunoblot was visualized using an enhanced chemiluminescence detection system (GE Healthcare life science).

### Plasmid construction and establishment of stable expression cell line

Mouse pre-miR-146a and pre-miR-222 gene were amplified from mouse peripheral blood lymphocyte genomic DNA. The DNA fragments were respectively cloned into the pLL3.7 vector (Promega, WI, USA) downstream of the U6 promoter. The DNA constructs were verified with DNA sequencing by BGI Life Tech Co. Ltd. (China). To produce the recombinant lentivirus, 293 T/17 cells were co-transfected with the vectors described above and with packaging plasmids using Lipofectamine 2000 according to the manufacturer’s guidelines. Infectious lentiviruses were harvested at 72 h post-transfection and filtered through 0.45-μm polyvinylidene fluoride filters. Recombinant lentiviruses were concentrated by ultracentrifugation (1.5 h at 80,000 g). The virus-containing pellet was dissolved in 1640 and stored at −80 °C. RAW264.7 cells were infected with concentrated virus in the presence of 10 μg/ml polybrene (Sigma-Aldrich, St. Louis, MO). To obtain cell lines stably expressing miR-146a and miR-222, RAW264.7 cells with GFP-expressing colonies were subsequently sorted by Flow cytometry (BD FAS Aria III Cell Sorter, Beckman Coulter) by Green Fluorescence Protein (GFP) expression to enrich the miR-146a and miR-222 expressing cells.

The precursor of p50 were amplified with specific primers by PCR from mouse poly(A)^+^ cDNA, and then cloned into the BamH I/EcoR I sites of pwpxl vector.

### Chromatin immunoprecipitation (ChIP) assay

RAW264.7 cells were cross-linked with 1% formaldehyde at room temperature for 8 min. Then removed formaldehyde and washed twice in cold PBS with protease inhibitor and harvested by centrifugation of 12,000 rpm for 10 min. Cells were lysed in SDS with protease inhibitor. Chromatin was sheared with a total time of 4 min 30 sec, ultrasound 10 sec gap 10 sec, 13,000 rpm centrifugal 10 min, then transferred the supernatant to a 2 ml new tube, the supernatant was diluted into 10X dilution of CHIP, 200 ul CHIP dilution was added to 1.8 ml of the supernatant to a final volume of 2 ml. Then 75 ul of Salmon Sperm DNA/Protein A Agarose-50% Slurry were added to eliminate the non-specific fragment, rotation for 30 minutes at 4 °C, centrifuged to collect the supernatant. The supernatant was incubated with the primary antibody (IgG or p50) at 4 °C for 14 h. To precipitate the antibody/antigen complex, 60 μl salmon sperm DNA/protein A agarose-50% slurry were added, and rating 1 h 4 °C, then centrifuged it at the speed of 1,000 rpm for 3 min at 4 °C. Removed the supernatant and eluted the precipitation with LiCl one time for 5 min and TE buffer for 5 min two times. The precipitation was mixed with 250 μl elution buffer (1%SDS, 0.1 M NaHCO_3_), rotated for 15 min and centrifugated for 12,000 rpm, 3 min. The combined eluates were de-crosslinked at 65 °C overnight with addition of 20 μl 5 M NaCl. DNA was extracted once with phenol/chloroform and precipitated with ethanol. DNA were washed once with 70% ethanol and resuspended in 10 μl TE buffer.

### Dual-Luciferase reporter assay

The cDNA of HEK293 cells was used to conduct conventional PCR to amplify the putative miR-222 binding site in CXCL12 3′-UTR and its mutated binding site. The wild-type 3′-UTR and the mutated 3′-UTR sequences of mouse CXCL12 were inserted into pMIR-report luciferase reporter vector (Promega, MI, USA) respectively, the constructed vector is named as CXCL12-WT and CXCL12-MUT. We cloned the complete sequence complementary to miR-222 into the pMIR-report vector as a positive control named as miR-222 reporter. The sequences were verified by DNA sequencing. HEK 293 cells were seeded in 24-well plates with 4 × 10^5^ cells per well. 14 hours later, the constructed vectors were co-transfected with phRL-TK (pMIR-report vector and phRL-TK transfected in a ratio of 10:1) and miR-222 mimics or scramble in HEK293 cells via Lipofectamine 2000 (Invitrogen). The luciferase activity was examined after transfected for 48 hours by using Modulus turner (microplore).

### Cell migration assay

5 × 10^4^ RAW264.7 cells were seeded onto the transwell migration upper chambers (8 μm pore size; Corning, Germany). Media containing 20% FBS or 4T1 culture medium was added to the lower chamber. After 24 hours, the non-migrating cells were removed with cotton wool, migration cells located on the lower surface of the chamber were stained with Giemsa stain and counted using a microscope (Leica, Germany). For siRNA and miRNA inhibitor transfection experiment, RAW264.7 cells were grown to confluence on 6-well plastic dishes and treated with indicated siRNA and/or miR-222 inhibitor. 24 hours after transfection, 5 × 10^4^ cells were seeded onto the transwell migration upper chambers. 4T1 culture medium was added to the lower chamber. A total of 5 areas were selected randomly from each well and the cells in three wells of each group were quantified. All the experiments were independently repeated three times.

### Statistics

The statistical analyses were performed using SPSS version 13.0 software. Student’s t tests were performed for comparison as described. Data are reported as the mean as mean ± SD. Significance is reported for values of P ≤ 0.05.

## Additional Information

**How to cite this article**: Li, Y. *et al.* Functions of miR-146a and miR-222 in Tumor-associated Macrophages in Breast Cancer. *Sci. Rep.* **5**, 18648; doi: 10.1038/srep18648 (2015).

## Supplementary Material

Supplementary Information
