# Supplementary material for: Functions of miR-146a and miR-222 in Tumor-associated Macrophages in Breast Cancer
Source: Sci Rep. 2015 Dec 22;5:18648. doi: 10.1038/srep18648 (PMC4686897; doi:10.1038/srep18648)
Supplement: Supplementary Information [file srep18648-s1.pdf]

Functions of miR-146a and miR-222 in Tumor-associated Macrophages in Breast Cancer

Yanshuang Li, Lianmei Zhao, Bianhua Shi, Sisi Ma, Zhenbiao Xu, Yehua Ge, Yanxin Liu, Dexian Zheng, Juan Shi

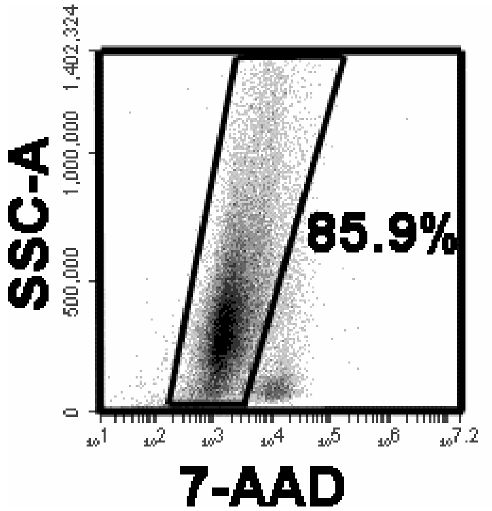

Supplementary Figure 1. Cell viability of TAMs purified from mice 4T1 tumors was analyzed by FACS using 7-AAD staining.

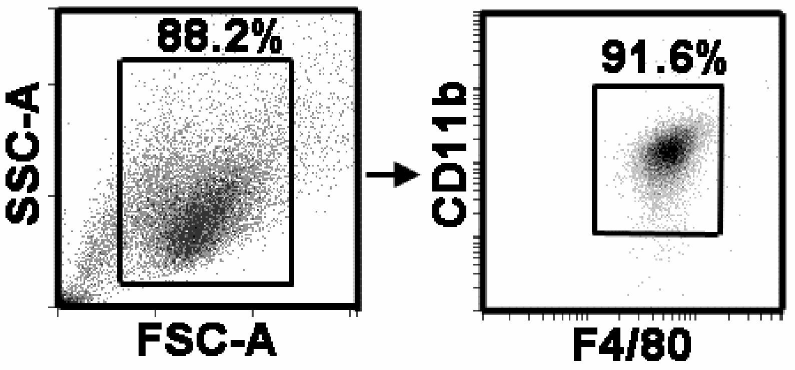

Supplementary Figure 2. Gating strategy used to identify total F4/80^{+} CD11b^{+} cells among TAMs purified from mice 4T1 tumors by FACS.

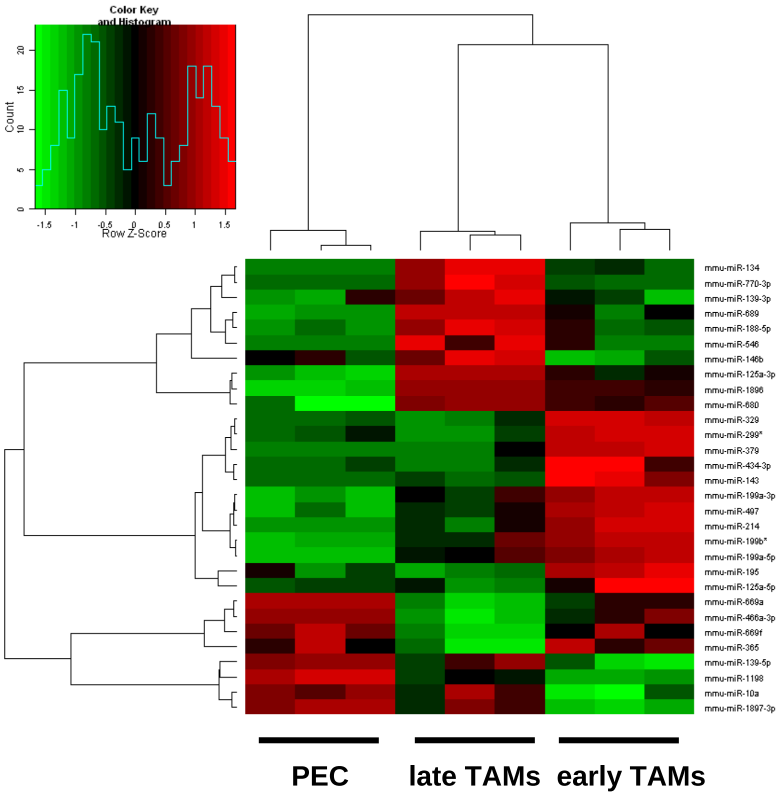

Supplementary Figure 3. Heatmap showing expression array data from the miRNA expression screening between early and late tumor TAMs (fold changes ≥ 2 or ≤ 0.5, p ≤ 0.05. Early TAM group refers to TAMs isolated from early 4T1 xenograft tumor (grow to 12 days) tissue. Late TAM group refers to TAMs isolated from advanced 4T1 xenograft tumor (grow to 25 days) tissue. Each sample is biologically duplicated.

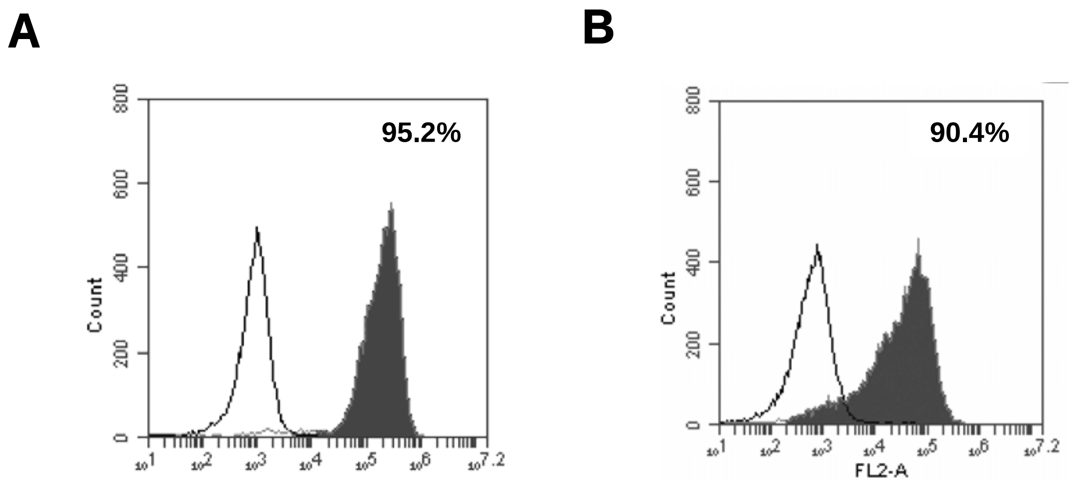

Supplementary Figure 4. TAMs purified from patients tumors (A) and PBMCs (B) were stained with anti-F4/80-PE then analyzed by FACS.

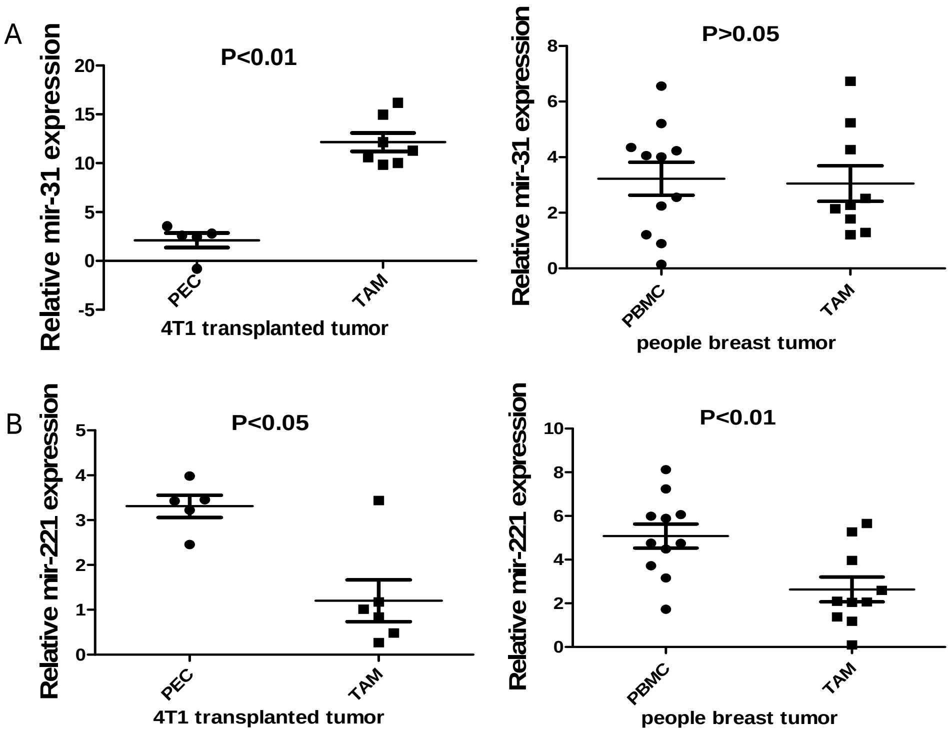

Supplementary Figure 5. Validation of the miRNAs microarray results in TAMs in mouse 4T1 transplanted tumor tissue and patients. A. MiR-31 was increased in TAMs from mice 4T1 transplanted tumor or in TAMs from breast cancer tissue compared with paired PBMC by qRT-PCR analysis. **B.** MiR-221 was decreased in TAMs from mice 4T1 transplanted tumor or in TAMs from breast cancer tissue compared with paired PBMC by qRT-PCR analysis. U6 snRNA was as the internal control .Error bars denote SD.

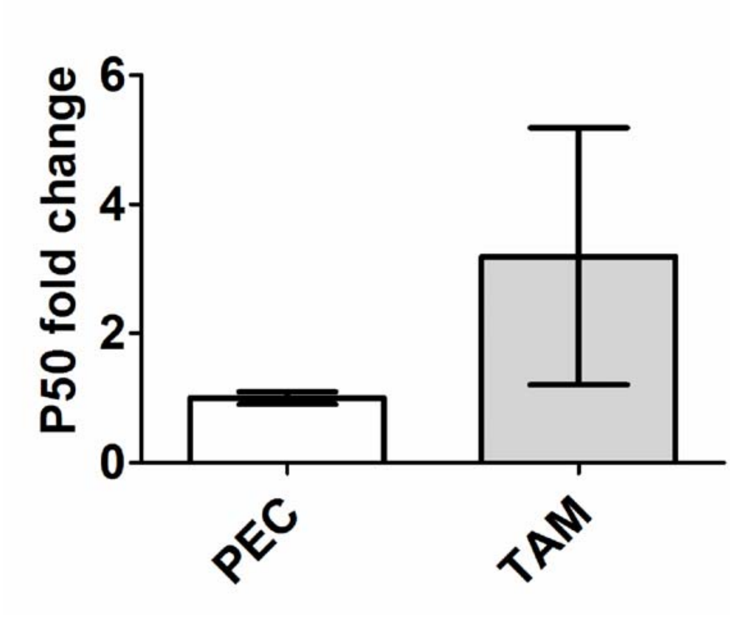

Supplementary Figure 6. qRT-PCR analysis of NF-κB p50 expression in TAMs from 4T1 xenograft tumors compared with PEC. U6 snRNA was as the internal control. n=3.

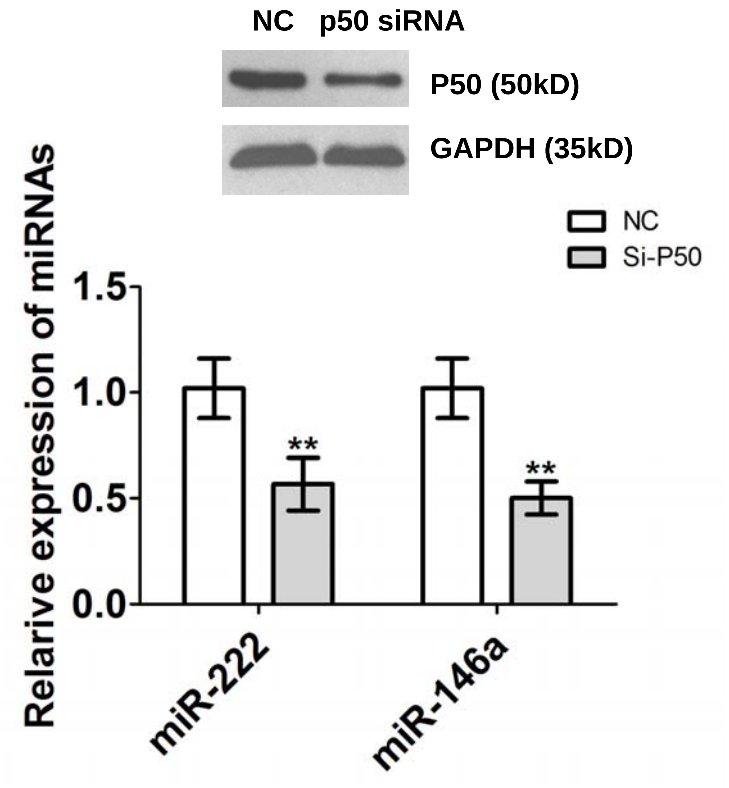

Supplementary Figure 7. qRT-PCR analysis of miR-146a and miR-222 expression levels in p50 knockdown RAW264.7 cells stimulated by IL-4 (50 ng/ml) for 12 h . U6 snRNA was used as an internal control. Mean±SD were obtained from three independent experiments. **, $p<0.01$.

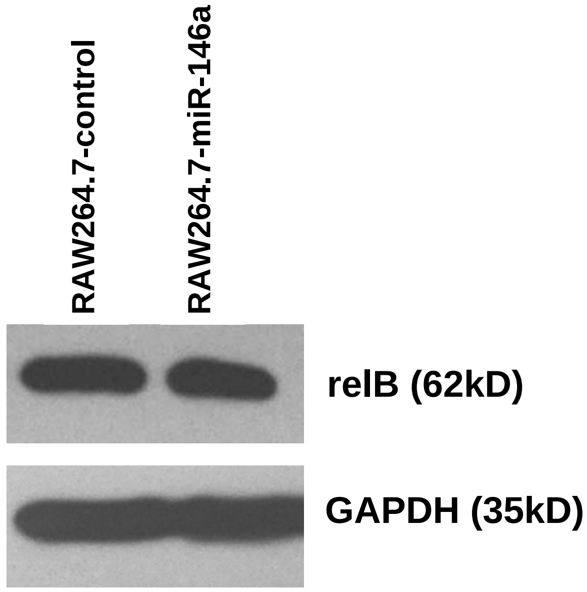

Supplementary Figure 8. Western blot assay of relB expression in RAW264.7 cells transfected with miR-146a inhibitor.

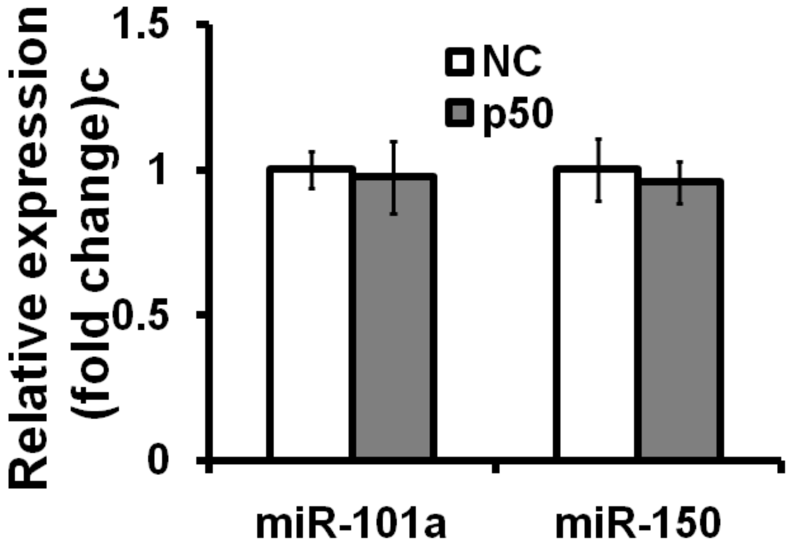

Supplementary Figure 9. qRT-PCR analysis of miR-146a and miR-222 expression levels in p50 overexpressing RAW264.7 cells. U6 snRNA was used as an internal control. Mean±SD were obtained from three independent experiments.

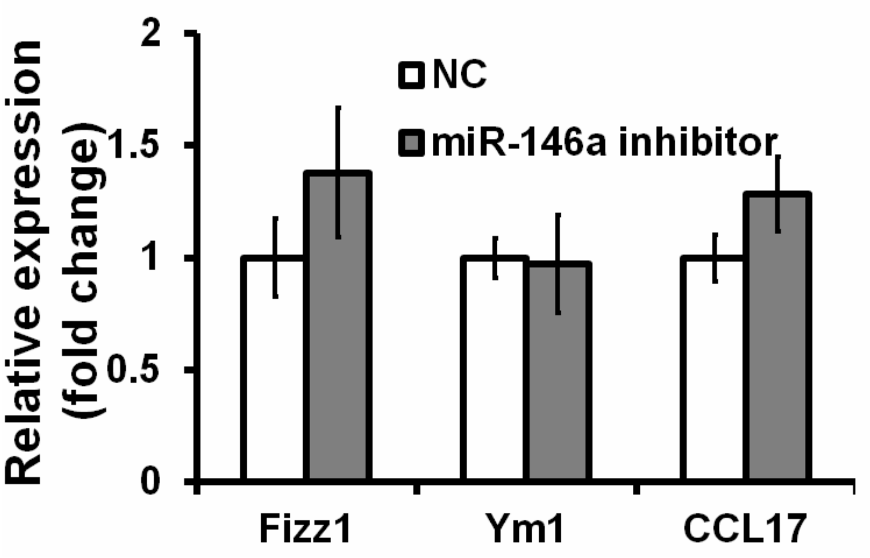

Supplementary Figure 10. qRT-PCR analysis of the expression of Fizz1, Ym1 and CCL17 in PECs transfected with the miR-146a inhibitor for 24 h and stimulated by IL-4 (50 ng/ml) for 12 h compared with the control group transfected with the NC inhibitor. β-Actin was used as an internal control. Mean±SD were obtained from three independent experiments.

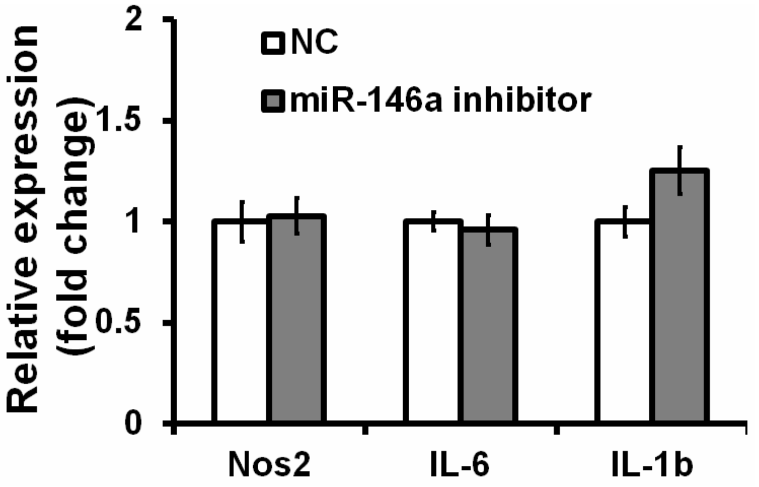

Supplementary Figure 11. qRT-PCR analysis of the expression of Nos2, IL-6 and IL-1b in PECs transfected with the miR-146a inhibitor for 24 h and stimulated by IL-4 (50 ng/ml) for 12 h compared with the control group transfected with the NC inhibitor. β-Actin was used as an internal control. Mean±SD were obtained from three independent experiments.

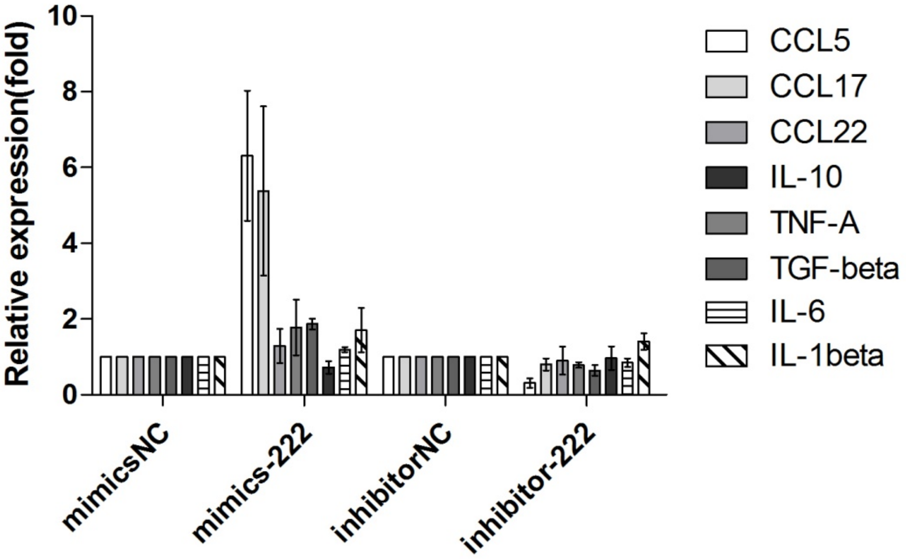

Supplementary Figure 12. Relative expression of cytokines were not changed significantly in PECs after transfected with miR-222 mimics or inhibitor for 24 h and stimulated by LPS (100 ng/ml) for 12h by qRT-PCR analysis. β-actin was used as an internal control. Mean±SD were obtained from three independent experiments.

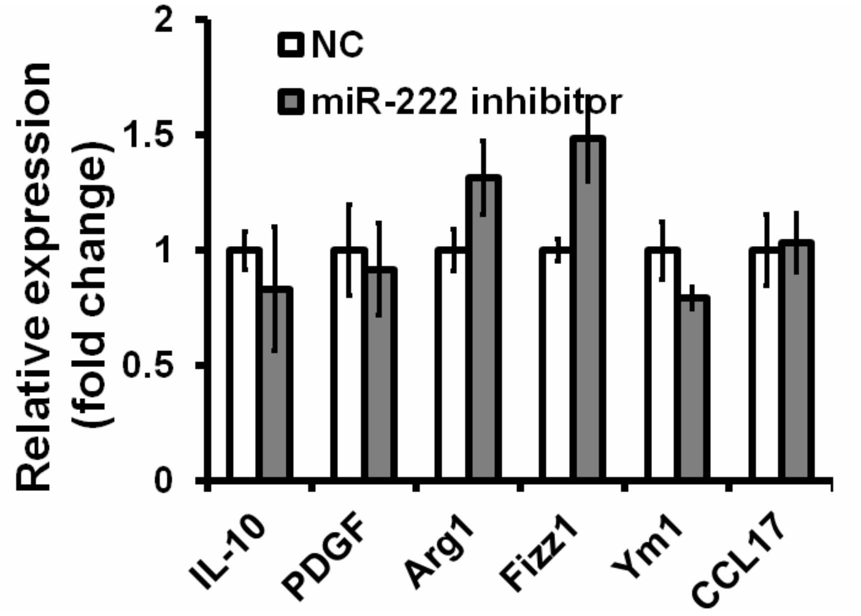

Supplementary Figure 13. qRT-PCR analysis of the expression of indicated mRNA in PECs transfected with the miR-222 inhibitor for 24 h and stimulated by IL-4 (50 ng/ml) for 12 h compared with the control group transfected with the NC inhibitor. β-Actin was used as an internal control. Mean±SD were obtained from three independent experiments.

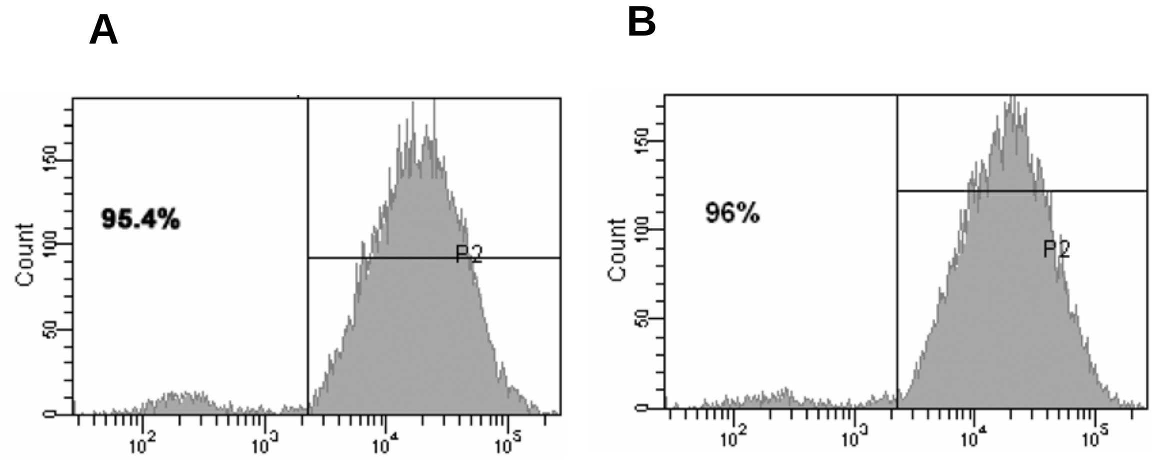

Supplementary Figure 14. Selection of RAW264.7 cells stably overexpressed miR-222. **(A)** RAW264.7 cells were stably transduced with lentivirus pLL3.7 and GFP-positive cells were sorted with FACS. **(B)** RAW264.7 cells were stably transduced with lentivirus pLL3.7-miR-222 and GFP-positive cells were sorted with FACS.

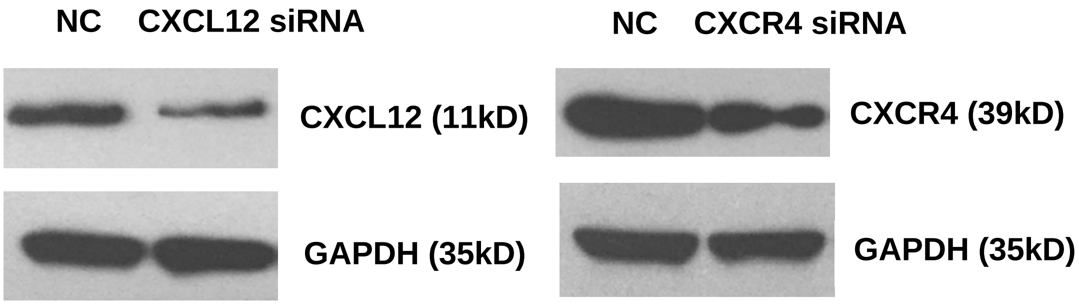

Supplementary Figure 15. Western blot assay of CXCL12 and CXCR4 expression in RAW264.7 cells transfected with siRNA against CXCL12 or CXCR4.

Supplementary Table 1. Differentially expressed miRNAs in late TAM compared with PEC (fold changes ≥ 2 or ≤ 0.5, p ≤ 0.05).

Gene ID	P-values	Fold change	regulation
mmu-miR-146a	0.000101	0.034095	Down
mmu-miR-222	0.000233	0.087006	Down
mmu-miR-29c*	7.03E-05	0.09445	Down
mmu-miR-467a-1*	0.00712	0.111557	Down
mmu-miR-378*	0.000656	0.129464	Down
mmu-miR-342-5p	0.009314	0.130845	Down
mmu-miR-150	0.003346	0.135758	Down
mmu-miR-24-1*	0.000167	0.139052	Down
mmu-miR-503	0.005072	0.145483	Down
mmu-miR-505	0.003081	0.150728	Down
mmu-miR-221	0.004844	0.151515	Down
mmu-miR-101a	0.000415	0.162955	Down
mmu-miR-7a*	0.002537	0.165298	Down
mmu-miR-467c	0.003615	0.188367	Down
mmu-miR-186	0.000456	0.18874	Down
mmu-miR-467a	0.00404	0.193394	Down
mmu-miR-501-3p	0.004951	0.202256	Down
mmu-miR-18a*	0.008798	0.207632	Down
mmu-miR-7a	0.00102	0.218135	Down
mmu-miR-29a*	0.004356	0.224273	Down
mmu-miR-450a-5p	0.002341	0.236806	Down
mmu-miR-1198	0.00117	0.281465	Down
mmu-miR-328	0.00141	0.283455	Down
mmu-miR-669f	0.002768	0.292397	Down
mmu-miR-484	0.008689	0.292769	Down
mmu-miR-324-3p	0.008436	0.300831	Down
mmu-miR-29a	0.006281	0.330012	Down
mmu-miR-29c	0.015491	0.350415	Down
mmu-miR-24	0.00433	0.354045	Down
mmu-miR-674	0.008678	0.360117	Down

mmu-miR-148a	0.001954	0.373219	Down
mmu-miR-21	0.00516	0.38883	Down
mmu-miR-29b	8.50E-05	0.41653	Down
mmu-miR-338-3p	0.002987	0.434946	Down
mmu-miR-322	0.007278	0.460058	Down
mmu-miR-142-5p	0.004332	0.461379	Down
mmu-miR-31	0.00536	83.70789	Up
mmu-miR-877	0.002515	83.19082	Up
mmu-miR-290-5p	0.000751	39.21011	Up
mmu-miR-721	0.002214	38.50123	Up
mmu-miR-135a*	0.00881	29.25945	Up
mmu-miR-483	0.003543	28.5149	Up
mmu-miR-714	0.003514	28.44157	Up
mmu-miR-370	0.000207	23.70778	Up
mmu-miR-1896	2.67E-05	21.95832	Up
mmu-miR-126-3p	0.008026	15.80166	Up
mmu-miR-1224	0.001299	11.20245	Up
mmu-miR-710	0.009483	10.64708	Up
mmu-miR-1892	0.000353	9.924646	Up
mmu-miR-705	0.004492	9.360324	Up
mmu-miR-134	0.006344	7.989754	Up
mmu-miR-1196	0.00915	6.743582	Up
mmu-miR-671-5p	0.005554	6.019463	Up
mmu-miR-1895	0.008733	5.610371	Up
mmu-miR-874	0.000963	4.576521	Up
mmu-miR-188-5p	0.004254	4.124566	Up
mmu-miR-689	0.001328	3.757902	Up
mmu-miR-132	0.003332	3.099332	Up
mmu-miR-1904	0.003869	2.466044	Up

Supplementary Table 2. Differentially expressed miRNAs in early TAM compared with PEC (fold changes ≥ 2 or ≤ 0.5, p ≤ 0.05).

Gene ID	P-values	Fold change	regulation
mmu-miR-31	0.00374	0.004973	Down
mmu-miR-96	0.000946	0.021484	Down
mmu-miR-183	0.003239	0.024403	Down
mmu-miR-126-3p	0.016934	0.027024	Down
mmu-miR-721	0.017507	0.03218	Down
mmu-miR-199a-5p	0.008091	0.033413	Down
mmu-miR-214	0.010354	0.035682	Down
mmu-miR-483	0.002421	0.036678	Down
mmu-miR-199a-3p	0.00082	0.048209	Down
mmu-miR-714	0.005222	0.053673	Down
mmu-miR-196b	0.005036	0.055366	Down
mmu-miR-130a	0.003975	0.05575	Down
mmu-miR-125b-5p	0.000192	0.059774	Down
mmu-miR-680	0.03168	0.062349	Down
mmu-miR-370	0.00028	0.073708	Down
mmu-miR-199b*	0.000251	0.076714	Down
mmu-miR-497	0.001448	0.079085	Down
mmu-miR-100	0.002614	0.081592	Down
mmu-miR-127	0.000443	0.086776	Down
mmu-miR-193b	0.000515	0.096506	Down
mmu-miR-31*	0.003089	0.101037	Down
mmu-miR-1896	2.29E-05	0.102621	Down
mmu-miR-143	0.021821	0.102721	Down
mmu-miR-1224	0.010747	0.112759	Down
mmu-miR-1897-5p	0.007363	0.11647	Down
mmu-miR-762	0.03495	0.11865	Down
mmu-miR-1892	0.023668	0.120144	Down
mmu-miR-181d	0.00056	0.130661	Down
mmu-miR-200c	0.008966	0.136508	Down
mmu-miR-141	0.020959	0.142112	Down

mmu-miR-574-5p	0.009358	0.154067	Down
mmu-miR-135a*	0.03699	0.155104	Down
mmu-miR-9	0.031592	0.158556	Down
mmu-miR-705	0.007579	0.162079	Down
mmu-miR-1187	0.002185	0.164663	Down
mmu-miR-379	0.008589	0.175642	Down
mmu-let-7b	0.000449	0.191704	Down
mmu-miR-125a-3p	0.04464	0.214899	Down
mmu-let-7c	0.000482	0.224544	Down
mmu-miR-1895	0.043925	0.230549	Down
mmu-let-7e	0.008476	0.234227	Down
mmu-miR-434-3p	0.040425	0.240722	Down
mmu-miR-329	0.001113	0.243662	Down
mmu-miR-299*	0.002342	0.244892	Down
mmu-miR-429	0.013693	0.278481	Down
mmu-miR-669n	0.013011	0.304409	Down
mmu-miR-466c-5p	0.020911	0.322001	Down
mmu-miR-152	0.000759	0.323208	Down
mmu-miR-337-5p	0.044336	0.411214	Down
mmu-miR-92a	0.002773	0.413223	Down
mmu-miR-195	0.019137	0.4133	Down
mmu-miR-24-2*	0.006873	20.21205	Up
mmu-miR-146a	0.001061	15.67881	Up
mmu-miR-326	9.63E-05	12.7344	Up
mmu-miR-29c*	0.000583	12.5754	Up
mmu-miR-342-5p	0.007117	10.50714	Up
mmu-miR-501-3p	0.000218	10.08778	Up
mmu-miR-222	0.022423	8.82095	Up
mmu-miR-194	0.00173	8.374756	Up
mmu-miR-532-3p	0.03127	8.008771	Up
mmu-miR-1198	8.74E-05	7.868306	Up
mmu-miR-24-1*	7.88E-06	7.842046	Up
mmu-miR-340-5p	0.042632	7.582957	Up
mmu-miR-7a*	0.008486	7.2986	Up

mmu-miR-342-3p	0.046903	7.151639	Up
mmu-miR-503	0.005224	7.115263	Up
mmu-miR-192	0.018988	7.030542	Up
mmu-miR-221	0.000894	6.705448	Up
mmu-miR-505	0.003086	6.631635	Up
mmu-miR-362-3p	0.007294	6.349561	Up
mmu-miR-340-3p	0.034466	6.155609	Up
mmu-miR-744	0.027029	5.141216	Up
mmu-miR-22*	0.000997	5.06572	Up
mmu-miR-148b	0.000118	4.912877	Up
mmu-miR-7a	0.002763	4.893464	Up
mmu-miR-467a-1*	0.011322	4.720779	Up
mmu-miR-30e*	0.007277	4.64424	Up
mmu-miR-185	0.004427	4.452607	Up
mmu-miR-29a*	0.004215	4.426796	Up
mmu-miR-128	0.012031	4.374172	Up
mmu-miR-532-5p	0.040119	4.303505	Up
mmu-miR-29c	0.006161	4.288416	Up
mmu-miR-484	0.001447	4.217105	Up
mmu-miR-24	0.004578	4.125112	Up
mmu-miR-350	0.023153	3.91565	Up
mmu-miR-34a	0.035697	3.759133	Up
mmu-miR-324-3p	0.000975	3.740098	Up
mmu-miR-450a-5p	0.001079	3.649161	Up
mmu-miR-700	0.044798	3.645213	Up
mmu-miR-29a	0.003888	3.58157	Up
mmu-miR-142-5p	0.030238	3.569847	Up
mmu-miR-139-5p	0.025426	3.502129	Up
mmu-miR-92b	0.021566	3.459879	Up
mmu-miR-21	0.002255	3.334121	Up
mmu-miR-29b	0.002913	3.321309	Up
mmu-miR-27a	0.026558	3.284782	Up
mmu-miR-18a*	0.004088	3.266999	Up
mmu-miR-1897-3p	0.011474	3.116137	Up

mmu-miR-328	0.026049	2.964014	Up
mmu-miR-23a	0.010446	2.931426	Up
mmu-miR-30b	0.003148	2.877121	Up
mmu-miR-22	8.39E-07	2.862469	Up
mmu-miR-10a	0.031159	2.843934	Up
mmu-miR-467c	0.011709	2.833879	Up
mmu-miR-101b	0.010939	2.604558	Up
mmu-miR-715	0.014805	2.399198	Up
mmu-miR-361	0.028092	2.360943	Up
mmu-miR-669a	0.033401	2.32335	Up
mmu-miR-130b	0.047465	2.318821	Up
mmu-miR-140*	0.042007	2.179845	Up
mmu-miR-28	0.005457	2.15592	Up
mmu-miR-877*	0.026072	2.151427	Up
mmu-miR-15a	0.001881	2.128147	Up
mmu-let-7f*	0.039594	2.110067	Up
mmu-miR-140	0.006046	2.074977	Up

Supplementary Table 3. Differentially expressed miRNAs in early TAM compared with late TAM (fold changes ≥ 2 or ≤ 0.5, p ≤ 0.05).

Gene ID	P-values	Fold change	regulation
mmu-miR-134	0.002074	0.181103	Down
mmu-miR-546	0.036159	0.319541	Down
mmu-miR-125a-3p	0.017817	0.333767	Down
mmu-miR-188-5p	0.015203	0.364437	Down
mmu-miR-680	0.002073	0.388048	Down
mmu-miR-139-5p	0.043574	0.397325	Down
mmu-miR-10a	0.042232	0.421272	Down
mmu-miR-1897-3p	0.018412	0.440007	Down
mmu-miR-770-3p	0.038318	0.443216	Down
mmu-miR-1896	0.001918	0.443778	Down
mmu-miR-689	0.035326	0.44455	Down
mmu-miR-1198	0.003989	0.451538	Down
mmu-miR-146b	0.008741	0.45941	Down
mmu-miR-139-3p	0.029943	0.491916	Down
mmu-miR-214	0.010931	9.351526	Up
mmu-miR-143	0.01941	8.314042	Up
mmu-miR-299*	0.00338	5.510805	Up
mmu-miR-497	0.003739	4.60159	Up
mmu-miR-329	0.003523	4.515401	Up
mmu-miR-379	0.003929	4.512791	Up
mmu-miR-199a-5p	0.028365	4.246828	Up
mmu-miR-434-3p	0.032264	4.194145	Up
mmu-miR-199a-3p	0.028489	4.050228	Up
mmu-miR-466a-3p	0.033722	4.025437	Up
mmu-miR-195	0.009762	3.186939	Up
mmu-miR-669a	0.03647	3.133085	Up
mmu-miR-199b*	0.047101	3.044941	Up
mmu-miR-669f	0.035637	2.629271	Up
mmu-miR-125a-5p	0.04652	2.627256	Up
mmu-miR-365	0.008463	2.147183	Up

Supplementary Table 4. Oligonucleotide sequence of siRNA in this study

Name of siRNA	Oligonucleotide sequence
**si-p50**	
siP50-mmu-2872	Sense 5'- GCCUGUGUUCACAUCUGAUTT -3'
	Antisense 5'- AUCAGAUGUGAACACAGGCTT -3'
siP50-mmu-1471	Sense 5'- GCCAGCUUCCGUGUUUGUUTT -3'
	Antisense 5'- AACAAACACGGAAGCUGGCTT -3'
siP50-mmu-593	Sense 5'- CCAGAAAUACCACUGUCAATT -3'
	Antisense 5'- UUGACAGUGGUAUUUCUGGTT -3'
**si-CXCL12**	
siCXCL12-mmu-333	Sense 5'- GCAUUGACCCGAAAUUAAATT -3'
	Antisense 5'-UUUAAUUUCGGGUCAAUGCTT-3'
siCXCL12-mmu-358	Sense 5'- CCAAGAGUACCUGGAGAAATT -3'
	Antisense 5'- UUUCUCCAGGUACUCUUGGTT -3'
**siRNA –CXCR4**	
siCXCR4-mmu-96	Sense 5'- CGAUCAGUGUGAGUAUAUATT -3'
	Antisense 5'- UAUAUACUCACACUGAUCGTT -3'
siCXCR4-mmu-1083	Sense 5'- GCCUCAAGAUCCUUUCCAATT -3'
	Antisense 5'- UUGGAAAGGAUCUUGAGGCTT -3'

Supplementary Table 5. Primers for miRNAs reverse transcription used in this study

Gene	Primer Sequence
U6	5'-3' CGCTTCACGAATTTGCGTGTCAT
miR-146a	5'-3' GTCGTATCCAGTGCGTGTCGTGGAGTCGGCAATTGCACTGGA TACGACAACCCA
miR-222	5'-3' GTCGTATCCAGTGCGTGTCGTGGAGTCGGCAATTGCACTGGATACGACACCCAG
miR-877	5'-3' GTCGTATCCAGTGCGTGTCGTGGAGTCGGCAATTGCACTGGATACGACCCCTGC
miR-31	5'-3' GTCGTATCCAGTGCGTGTCGTGGAGTCGGCAATTGCACTGGATACGACCAGCTA
miR-221	5'-3' GTCGTATCCAGTGCGTGTCGTGGAGTCGGCAATTGCACTGGATACGACGAAACC

Supplementary Table 6. Primers Sequence used in this study

Gene	Primer Sequence
U6	5'-3'GCTTCGGCAGCACATATACTAAAAT<br>5'-3'CGCTTCACGAATTTGCGTGTCAT
miR-146a	5'-3'GGGGGGGGTGAGAACTGAA
miR-31	5'-3'GGGGGGGGAGGCAAGATGC
miR-877	5'-3'GGGGGGGGTAGAGGAGATG
miR-221	5'-3'GGGGGGGGGAGCTACATTGTC
miR-222	5'-3'GGGGGGGGGAGCTACATCTGG
miRNA universal primer	5'-3'GGGGGGGGTAGAGGAGATG
β-actin	5'-3'CATGTACGTTGCTATCCAGGC<br>5'-3'CTCCTTAATGTCACGCACGAT
Arg1	5'-3' CTTGGCTTGCTTCGGAACTC<br>5'-3' GGAGAAGGCGTTTGCTTAGTTC
IL-12	5'-3' ATCTACCGAAGTCCAATGCAA<br>5'-3' ATTTCAACAGCATAAGGCCAA
IL-10	5'-3' CATACTGCTAACCGACTCCT<br>5'-3' CTCCACTGCCTTGCTCTTA
IL-1β	5'-3' ATCTCGCAGCAGCACATC<br>5'-3' CAGCAGGTTATCATCATCATCC
IL-6	5'-3' CAGAAGGAGTGGCTAAGGACCA

	5’-3’ ACGCACTAGGTTTGCCGAGTAG
Nos2	5’-3’ GTTCTCAGCCCAACAATACAAGA<br><br>5’-3’ GTGGACGGGTCGATGTCAC
P50	5’-3’ CGGGATCCCACCATGGCAGACGATG ATCCCTAC<br><br>5’-3CGGAATTCCTAAACCACCCAGGTACCTTTG
CCL5	5’-3’ ATATGGCTCGGACACCACTC<br><br>5’-3’ GTGACAAACACGACTGCAAGA
CCL22	5’-3’ AGGGAGGAGGACCTGATGAC<br><br>5’-3’ GGTAAGGCTGGCCTGAATGT
CCL17	5’-3’ GCTCTGCTTCTGGGGACTTT<br><br>5’-3’ GGGTCTGCACAGATGAGCTT
TNFα	5’-3’ GCCACCACGCTCTTCTGTCTAC<br><br>5’-3’ GGCTACAGGCTTGTCACTCGAA
PDGF	5’-3’ ACCAGGACGGTCATTTACG<br><br>5’-3’ TGATTCCCTACGCCTTCC
Ym1	5’-3’ CATGAGCAAGACTTGCGTGAC<br><br>5’-3’ GGTCCAAACTTCCATCCTCCA
Fizz1	5’-3’ TCCCAGTGAATACTGATGAGA<br><br>5’-3’ CCACTCTGGATCTCCCAAGA-3
Mcp-1	5’-3’ TTG ACC CGT AAA TCT GAA GCT AAT<br><br>5’-3’ TCA CAG TCC GAG TCA CAC TAG TTC AC

Mgl-2	5’-3’GATAACTGGCATGGACATATG<br><br>5’-3’TTTCTAATCACCATAACACATTC
